# Layered Double Hydroxides: A Novel Promising 2D Nanomaterial for Bone Diseases Treatment

**DOI:** 10.1002/advs.202301806

**Published:** 2023-06-17

**Authors:** Yixin Bian, Xuejie Cai, Zehui Lv, Yiming Xu, Han Wang, Chaoliang Tan, Ruizheng Liang, Xisheng Weng

**Affiliations:** ^1^ Department of Orthopedic Surgery State Key Laboratory of Complex Severe and Rare Diseases Peking Union Medical College Hospital Chinese Academy of Medical Science and Peking Union Medical College Beijing 100730 P. R. China; ^2^ Department of Chemistry and Center of Super‐Diamond and Advanced Films (COSDAF) City University of Hong Kong Kowloon Hong Kong P. R. China; ^3^ Shenzhen Research Institute City University of Hong Kong Shenzhen 518057 P. R. China; ^4^ State Key Laboratory of Chemical Resource Engineering Beijing Advanced Innovation Center for Soft Matter Science and Engineering Beijing University of Chemical Technology Beijing 100029 P. R. China

**Keywords:** 2D nanomaterial, bone disease, bone tissue engineering, layered double hydroxides

## Abstract

Bone diseases including bone defects, bone infections, osteoarthritis, and bone tumors seriously affect life quality of the patient and bring serious economic burdens to social health management, for which the current clinical treatments bear dissatisfactory therapeutic effects. Biomaterial‐based strategies have been widely applied in the treatment of orthopedic diseases but are still plagued by deficient bioreactivity. With the development of nanotechnology, layered double hydroxides (LDHs) with adjustable metal ion composition and alterable interlayer structure possessing charming physicochemical characteristics, versatile bioactive properties, and excellent drug loading and delivery capabilities arise widespread attention and have achieved considerable achievements for bone disease treatment in the last decade. However, to the authors' best knowledge, no review has comprehensively summarized the advances of LDHs in treating bone disease so far. Herein, the advantages of LDHs for orthopedic disorders treatment are outlined and the corresponding state‐of‐the‐art achievements are summarized for the first time. The potential of LDHs‐based nanocomposites for extended therapeutics for bone diseases is highlighted and perspectives for LDHs‐based scaffold design are proposed for facilitated clinical translation.

## Graphical abstract

In this work, for the first time, the advantages of LDHs for treating orthopedic disorders are outlined and the corresponding state‐of‐the‐art achievements are summarized. The potentials of LDHs‐based nanocomposites for extended therapeutics for bone diseases and perspectives of LDHs‐based scaffold design for facilitated clinical translation are also highlighted.

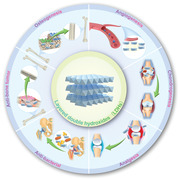

## 1 Introduction

Bone consisting of bone cells embedded in hierarchical intercellular collagen and calcium phosphate plays an important role in the body structural support, organs protection, and endocrine system and hematopoiesis regulation.^[^ ^1^, ^2^ ^]^ Bone diseases including bone defects, bone infections, osteoarthritis, and bone tumors seriously affect the life quality of the patient and bring serious economic burdens to social health management.^[^ ^3^, ^4^, ^5^ ^]^ However, the current clinical treatment effect for these diseases are far away from satisfactory.

During the last decades, material science and nanotechnology have made a significant impact on the therapies for bone diseases.^[^ ^6^, ^7^ ^]^ For example, bone tissue engineering aiming to combat the limitations of conventional treatments for bone defects has emerged as a promising field, where biomaterials‐based strategies account for an essential component.^[^ ^8^, ^9^, ^10^, ^11^ ^]^ Various materials have been exploited and achieved encouraging results in bone tissue engineering, such as ceramics, polymers, and metals.^[^ ^12^, ^13^, ^14^ ^]^ For bone infections and bone tumors, materials with intrinsic antibacterial or antitumor properties or loaded with antibacterial or antitumor drugs have also been designed for effective bacterial and tumor elimination and functional reconstruction of the affected bone.^[^ ^15^, ^16^, ^17^, ^18^ ^]^ Various hydrogel microspheres with anti‐inflammatory and chondrogenic properties have also been developed to alleviate inflammatory pain and halt cartilage degeneration in osteoarthritis patients.^[^ ^19^, ^20^, ^21^ ^]^ Nevertheless, the therapeutic efficiency of these biomaterial‐based strategies for bone diseases is far away from excellent and still has a long way to go before entering clinical practice.^[^ ^9^, ^11^, ^22^ ^]^

**Figure 1:**
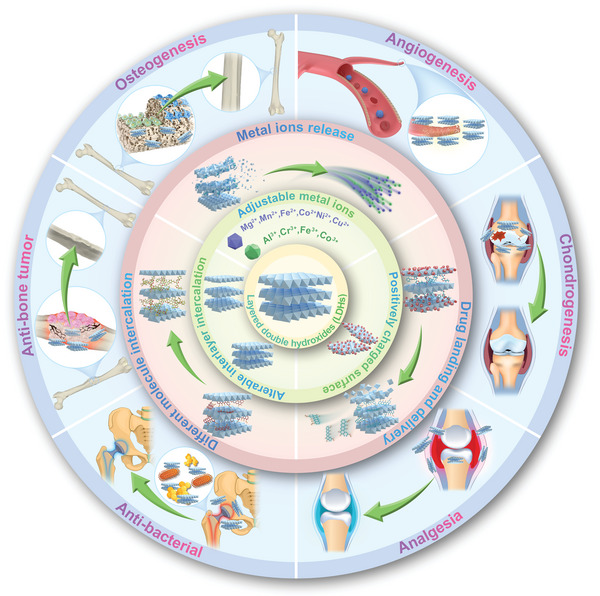
2D LDHs with charming physicochemical characteristics and versatile bioactive properties is a promising candidate for multiple bone disease treatment.

Layered double hydroxides (LDHs) composed of positively charged metallic layers and interlayer anions possess great biocompatibility and unique physicochemical properties have recently attracted tremendous attention in biomedical field.^[^ ^23^, ^24^, ^25^ ^]^ Originally, LDHs were used as active ingredients in antacids and anti‐pepsin agents to relieve stomach pain caused by excess gastric acid and achieved good curative effects.^[^ ^25^, ^26^ ^]^ The pioneering study applying LDHs for bone disease treatment was first explored by Lin et al.^[^ ^27^ ^]^ in 2011, who directly grew oriented MgFe‐LDHs on pure Mg substrate to obtain better osteoinduction and osteointegration performance of Mg substrate. It turned out MgFe‐LDHs‐Mg substrate possesses improved hydrophilicity, better cellular spreading, and cellular interaction behavior, as well as much higher corrosion resistance compared with pure Mg. Since then, LDHs with adjustable metal ions composition possessing charming physicochemical characteristics (positively charged surface, alterable interlayer structure, etc.), versatile bioactive properties (osteogenesis, angiogenesis, chondrogenesis, etc.), and excellent substance loading and delivery capabilities (drug, bioactive molecule, photosensitizer, etc.) have arose widespread attention and achieved great achievements in bone diseases treatment (**Figure** **1**).^[^ ^28^, ^29^, ^30^, ^31^, ^32^, ^33^, ^34^, ^35^, ^36^ ^]^ However, to our best knowledge, no review has comprehensively summarized the advances of LDHs in treating bone diseases so far. Herein, we outline the advantages of LDHs‐based strategies for treating bone diseases and summarize the corresponding state‐of‐the‐art achievements for the first time. We also highlight the potential of LDHs for extended orthopedic applications and propose some perspectives for LDHs‐based scaffold design for facilitated clinical translation.

## 2 The Structure and Preparation of LDHs

**Figure 2:**
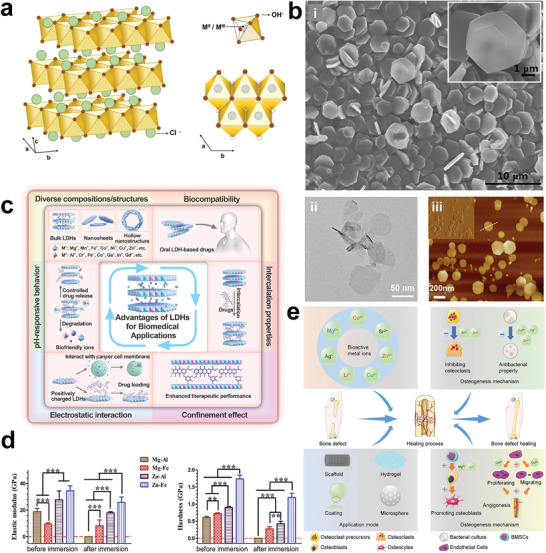
a) Schematic diagram of LDH structures. M^2+^ ions and M^3+^ ions positioned in octahedral [M(OH)_6_] units share edges in a layered fashion. The layers are in [ab] plane while assembling of the layers is accomplished in the [c] axis direction. Reproduced with permission.^[^ ^39^ ^]^ Copyright 2018, John Wiley and Sons. b) The SEM (i), TEM (ii), and AFM (iii) images of hexagonal LDHs nanosheets. Reproduced with permission.^[^ ^40^ ^]^ Copyright 2017, American Chemical Society. Copyright 2023, John Wiley and Sons. Copyright 2022, Awassa et al. c) Diagrammatic presentation of the characteristics and advantages of LDH‐based nanomaterials for biomedical applications. Reproduced with permission.^[^ ^25^ ^]^ Copyright 2022, Royal Society of Chemistry. d) Elastic modulus and hardness of different pressed pristine LDH discs before and after being immersed in the medium for 10 days. All values are similar to cortical bone. Reproduced with permission.^[^ ^54^ ^]^ Copyright 2018, Elsevier. e) Application mode and osteogenesis mechanism of bioactive metal ions in the healing process of bone defects. Reproduced with permission.^[^ ^55^ ^]^ Copyright 2022, Royal Society of Chemistry. SEM, scanning electron microscope; TEM, transmission electron microscopy; AFM, atomic force microscopy.

LDHs are a class of anionic layered clays containing hydroxide layers of divalent metal cations and trivalent metal cations, the formula of which is expressed as [M^2+^_1 −_ *_x_* M^3+^ *_x_* (OH)_2_](A*^n^* ^−^)*_x_*_/_ *_n_* ·mH_2_O, where M^2+^ and M^3+^ represent a bivalent metallic cation and a trivalent metallic cation and A*^n^* ^−^ is an interlayer anion.^[^ ^37^, ^38^ ^]^ Specifically, six hydroxide ions constitute an octahedral unit and are coordinated with centrally located metal cations, a hydroxide ion is also shared by three octahedral units (or three metal ions) to present the hexagonal shape of LDHs nanosheet (**Figure** **2**a,b).^[^ ^39^, ^40^, ^41^, ^42^ ^]^ The M^2+^/M^3+^ ratio commonly ranged from 2 to 4, of which LDHs can obtain better purity and crystallization.^[^ ^43^ ^]^ Notably, the metal ion composition, interlayer spacing and structure, specific surface area, surficial morphology of LDHs are alterable, which endow LDHs with different physicochemical characteristics and biological activities for enhanced biomedical application scenarios (Figure 2c).^[^ ^25^, ^44^, ^45^, ^46^, ^47^ ^]^

Regarding the synthesis of LDHs, “top‐down” and “bottom‐up” preparation techniques are the two most commonly applied methods. Typical, “top‐down” techniques usually employ physical shear forces or chemical intercalation to break up connections between neighboring layers from their blocky counterparts to obtain monolayers or multi‐nanolayers. Although miniaturizing bulk LDHs is a convenient process, the method relies on either removing or dividing the parent material, which leads to uneven surfaces. The bottom‐up strategy, in contrast, depends on the direct generation of LDH nanosheets utilizing chemical processes. The latter can be utilized to produce more intricate, flawless surfaces. Coprecipitation is a traditional bottom‐up procedure, with advantages of cheap, simple, and conducive to mass production. In this way, a salt solution containing divalent and trivalent metal cations is gently introduced into the target anion solution. With the addition of sodium hydroxide or urea, cationic hydroxides are simultaneously precipitated to provide an alkaline environment with a pH range of 6–11. A suitable temperature of 60–80 °C is also necessary for increasing the crystallinity of produced LDHs. For the hydrothermal synthesis method, alkaline solution and metal ions–contained solution are mixed in a hydrothermal reactor with high temperature and pressure, which can produces LDHs with outstanding crystallinity and high purity. Other typical methods involve anion exchange, atom economy method, and separate nucleation and aging steps, and so on. In addition to pristine LDH, LDH‐based nanomaterials for biomedical applications could be developed via a range of synthesis techniques such as reconstruction and in situ growth, and so on, to achieve diverse structures and varied physicochemical features.^[^ ^25^, ^48^, ^49^ ^]^

Moreover, as 2D nanomaterial, LDHs were also widely explored as coating material to promote the bioactivity of conventional scaffolds. In situ growth with high simplicity and efficiency is the most frequently employed approach to coat LDHs on magnesium‐based materials, which roughly includes in situ electrodeposition, hydrothermal treatment, urea hydrolysis, and steam coating. On the other hand, the adsorption process is a mass transfer strategy that involves the sorption of different species over specific surfaces using erratic forces of attraction. For example, positively charged LDHs tend to bond with negatively charged metal nanoparticles through non‐covalent interactions. Moreover, coprecipitation, electrochemical deposition, and anion exchange are also commonly used methods. The thickness of LDHs coating falls between 0.82 and 80 µm, which were typically adjustable according to the expected function exit by controlling the milder temperature or changing the ratio of metal ions. Meanwhile, the density of the LDHs coating could also be adjusted by altering the processing time and pH value during the fabrication process.^[^ ^49^, ^50^, ^51^ ^]^

## 3 The Advantages of LDHs in Treating Bone Diseases

**Figure 3:**
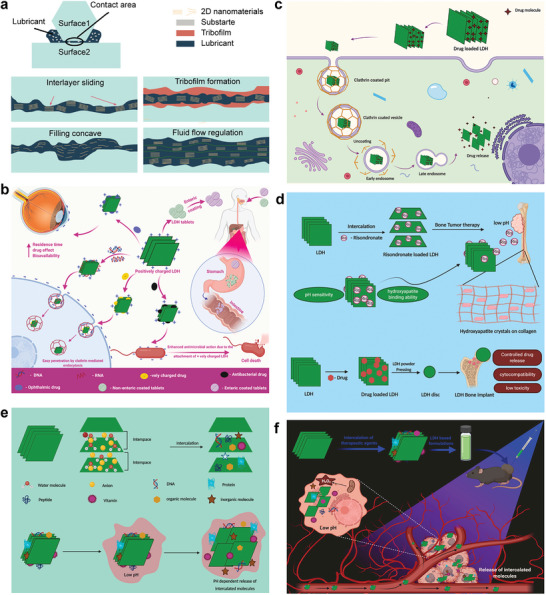
a) Schematic representation of the mechanism by which 2D LDHs functioned in lubrication. Improved lubrication greatly lowers the surface friction at interfaces. Reproduced with permission.^[^ ^72^ ^]^ Copyright 2018, Elsevier. b) The role of positive charge of LDHs in different substrates delivery applications. Reproduced with permission.^[^ ^81^ ^]^ Copyright 2021, Elsevier. c) Clathrin‐mediated endocytosis of LDH and intracellular drug release. Reproduced with permission.^[^ ^81^ ^]^ Copyright 2021, Elsevier. d) Therapy of bone tumor with risedronate intercalated LDHs and therapy of bone implantation with drug‐loaded LDHs. Reproduced with permission.^[^ ^81^ ^]^ Copyright 2021, Elsevier. e) Diagrammatic illustration of alkaline LDHs facilitates molecules insertion and release of LDHs intercalated molecules in low pH microenvironment. Reproduced with permission.^[^ ^81^ ^]^ Copyright 2021, Elsevier. f) Illustration on the possible reaction process of the drug released from LDHs at H_2_O_2_ rich and acidic tumor microenvironment. Reproduced with permission.^[^ ^81^ ^]^ Copyright 2021, Elsevier.

With adjustable chemical composition and unique physicochemical properties, LDH‐based nanomaterials hold many advantages in treating bone diseases as summarized in the following: 1) With great biocompatibility and negligible cytotoxicity, LDHs can be widely fabricated and explored as drugs, injections, and implant coating materials for bone diseases treatment.^[^ ^52^, ^53^ ^]^ 2) Pristine LDHs (Mg‐Al, Mg‐Fe, Zn‐Al, and Zn‐Fe) possess an elastic modulus of 9–35 GPa, which is similar to cortical bone (5–23 GPa) and can provide sufficient mechanical support but avoid stress shielding as bone‐repair material (Figure 2d).^[^ ^54^ ^]^ 3) The lamellar metal ions of LDHs are adjustable, of which different metal ions have been proven to possess the capability of regulating various pathophysiologic processes (Figure 2e).^[^ ^55^ ^]^ For example, Mg^2+^, Fe^3+^, and Ca^3+^ have been widely applied as osteogenic metal ions in the field of bone tissue engineering.^[^ ^56^, ^57^, ^58^, ^59^, ^60^ ^]^ Cu^2+^ and Sr^3+^ have been proven to possess potent angiogenesis properties, which is in favor of inducing vascularized bone regeneration.^[^ ^61^, ^62^, ^63^ ^]^ Mn^2+^ and high concentrations of Mg^2+^ showing chondrogenic properties can be utilized to delay cartilage degeneration in osteoarthritis patients.^[^ ^64^, ^65^, ^66^, ^67^ ^]^ Antibacterial metal ions including Zn^2+^ and Mn^2+^ can also be doped in LDHs and endow LDHs with favorable antibacterial properties.^[^ ^68^, ^69^, ^70^ ^]^ Thus, LDHs composed of different bioactive metal ions can propose versatile therapeutic effects and are promising in treating different bone diseases. 4) The ultra‐thin layer structure of LDHs and negligible shear strength between the layers make LDHs easy to enter the friction surfaces and transform sliding friction into rolling friction, thus significantly decreasing the friction coefficient between the surfaces,^[^ ^71^, ^72^, ^73^, ^74^ ^]^ which is promising to be applied in treating osteoarthritis that is characterized by increased friction in joints (**Figure** **3**a). 5) ROS generated by specific LDHs (e.g., Zn‐LDHs and Ti‐LDHs) with outstanding photocatalytic performance and abundant hydroxyl groups on the surface can be a potential strategy for bone infection and bone tumor treatment.^[^ ^42^, ^75^, ^76^, ^77^ ^]^ 6) LDHs possess superior substrate loading and delivery abilities with the following mechanism: i) The large specific surface area (100–600 m^2^ g^−1^) and alterable interlayer spacings (0.73–2.28 nm) endow LDHs with potent substrate loading and delivery ability.^[^ ^78^, ^79^, ^80^ ^]^ ii) Positively charged LDHs tend to conjugate with negatively charged substrates by electrostatic force and the abundant hydroxyl groups on LDHs surface can further enhance the substrates loading ability of LDHs (Figure 3b).^[^ ^81^, ^82^, ^83^, ^84^, ^85^, ^86^, ^87^ ^]^ iii) Positively charged LDHs tend to interact with the negatively charged cell surface protein (Clathrin) or bacterial membranes, making LDHs effective for intracellular and intrabacterial functional substrate delivery for enhanced therapeutic effects (Figure 3c),^[^ ^25^, ^81^, ^88^ ^]^ where the functional substrate can be drugs, bioactive molecules, genes, and photosensitizers, which allow LDHs to participate various physiological processes and exert corresponding therapeutic effects (Figure 3d).^[^ ^81^ ^]^ iv) The confinement effect of LDHs can maintain the stability and dispersibility of loaded drugs or other substrates by protecting them from degradation in vivo thus enhancing therapeutic efficiency.^[^ ^25^, ^89^ ^]^ v) Targeted drug delivery can also be realized due to alkaline LDHs degrading faster and releasing more drugs in acidic or H_2_O_2_‐riched microenvironment that is commonly observed in bone infections, bone tumors, and osteoarthritis (Figure 3e,f).^[^ ^81^, ^90^, ^91^, ^92^, ^93^ ^]^ With the above advantageous characteristics, LDHs can prominently outperform other porous biomaterials such as ceramics and polymers that are commonly used in orthopedics regarding drug loading and delivery. Taking alendronate (an anti‐osteoporosis drug) as an example, the loading content and encapsulation efficiency of MgAlYb‐LDHs for alendronate are significantly higher than that of other porous orthopedic biomaterials, as summarized in **Table** **1**. Thus, with superb loading capacity and target delivery ability determined by acid‐responsive degradation properties, LDHs are promising to serve as an effective drug loading and delivery platform for bone disease treatment.

**Table 1:** The loading content and encapsulation efficiency of different porous biomaterials for alendronate

Biomaterial	Category	LC (%)	EE (%)	Reference
MgAlYb‐LDHs	2D nanomaterial	197	98.6	[32]
Porous hydroxyapatite	Ceramic	21.7	92.3	[94]
Porous calcium phosphate	Ceramic	14.4	72	[95]
Chitosan nanoparticles	Natural polymer	/	66.8	[96]
Porous polycaprolactone	Synthetic polymer	20.7	34	[97]
PLGA/hydroxyapatite	Composite	/	36.2	[98]

LC, loading content; EE, encapsulation efficiency; LDHs, layered double hydroxides; PCL, polycaprolactone; PLGA, poly(lactic‐*co*‐glycolic acid).

## 4 LDHs for Bone Tissue Engineering

Bone tissue engineering aims to design and fabricate biomaterial‐based scaffolds that outperform autologous bone grafts to effectively treat bone defects by providing temporary mechanical support during bone‐repair process and inducing mineralized matrix deposition.^[^ ^99^, ^100^, ^101^ ^]^ Appropriate selection of biomimetic materials is essential for effective and successful bone regeneration and remolding. First, appropriate mechanical strength is important for bone tissue engineering materials to provide sufficient support and avoid stress‐shielding effects during the bone‐repair process.^[^ ^102^, ^103^, ^104^ ^]^ Second, good biocompatibility and negligible cytotoxicity are prerequisites for materials applied in bone tissue engineering.^[^ ^11^ ^]^ Third, the hypothetical materials should possess favorable osteogenesis, osteoinduction, and osteointegration properties to induce new bone formation and integrate neo‐bone with the implanted scaffold.^[^ ^103^, ^105^, ^106^ ^]^ Fourth, angiogenesis properties of materials also greatly matter because neovascularization in bone defect areas can facilitate oxygen and nutrition supply as well as metabolite elimination thus promoting bone regeneration.^[^ ^107^, ^108^, ^109^ ^]^ Finally, materials with extra chondrogenesis properties are idealistic in dealing with osteochondral defects, articular cartilage defects, and osteoarthritis.^[^ ^110^, ^111^, ^112^ ^]^ Since the 1990s, biomaterials including ceramics, natural polymers, synthetic polymers, metals, and carbon‐based materials attract increasing attention in bone tissue engineering.^[^ ^102^, ^113^, ^114^, ^115^, ^116^ ^]^ However, all the above biomaterials bear some disadvantages hindering clinical translation. For example, ceramics are commonly brittle and tend to suffer prosthetic fractures. Natural polymers bear poor mechanical properties and cannot provide sufficient support for bone healing. Synthetic polymers and carbon‐based materials are criticized for potential biotoxicity. Metals tend to induce stress shielding that is detrimental to bone reconstruction due to excessive mechanical properties. Therefore, LDHs with good biocompatibility and biomechanical properties, versatile bioactivity, and excellent drug loading and delivery ability have emerged as promising candidates for bone tissue engineering in the last decade. The comparison of LDHs with other conventional orthopedic materials in terms of several most important properties for bone tissue engineering is summarized in **Table** **2**.

**Table 2:** The comparison of LDHs with other conventional bone tissue engineering materials

Category	Examples	Biocompatibility	Mechanical property	Biodegradability	Osteogenesis	Angiogenesis	Chondrogenesis	Reference
LDHs	MgAl‐LDH	Good	Excellent	Good	Excellent	Good	Good	[36, 117, 118, 119]
Ceramic	Hydroxyapatite, bioactive glass	Good	Excellent	Poor	Good	Poor	Poor	[12, 120, 121, 122]
Natural polymer	Collagen, hydrogel	Excellent	Poor	Excellent	Fair	Poor	Poor	[123, 124, 125]
Synthetic polymer	PCL, PLA, PMMA	Poor	Fair	Poor	Poor	Poor	Poor	[13, 126, 127, 128]
Metals	Titanium, magnesium alloy	Good	Good	Poor	Fair	Poor	Poor	[14, 129, 130, 131]
Carbon‐based nanomaterials	Graphene, carbon nanotubes	Fair	Excellent	Fair	Good	Poor	Poor	[132, 133, 134]

LDHs, layered double hydroxides; PCL, polycaprolactone; PLA, polylactic acid; PMMA, polymethyl methacrylic.

### 4.1 The Mechanical, Corrosion Resistance, and Degradation Properties of LDHs

**Figure 4:**
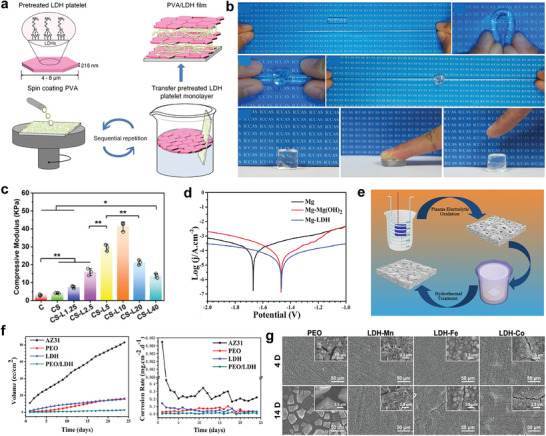
a) Schematic diagram of fabrication of bioinspired multilayered PVA/LDH hybrid films. Reproduced with permission.^[^ ^138^ ^]^ Copyright 2014, American Chemical Society. b) The prepared LDH/PAM hydrogel can withstand various mechanical deformations such as elongation, bending, knotting, knotting and subsequent elongation, and compression. Reproduced with permission.^[^ ^139^ ^]^ Copyright 2015, Royal Society of Chemistry. c) The compressive modulus of various CS samples. Reproduced with permission.^[^ ^117^ ^]^ Copyright 2022, John Wiley and Sons. d) Potentiodynamic polarization of Mg, Mg‐Mg(OH)_2_, Mg‐LDH, and accumulative hydrogen evolution of various samples after immersed in PBS. Reproduced with permission.^[^ ^34^ ^]^ Copyright 2021, Cheng et al. e) Schematic illustration of the preparation of PEO/MgAl‐LDH composite coating. Reproduced with permission.^[^ ^146^ ^]^ Copyright 2017, Springer Nature. f) Hydrogen evolution and corrosion rate of all samples. Reproduced with permission.^[^ ^146^ ^]^ Copyright 2017, Springer Nature. g) Corrosion morphologies of PEO, LDH‐Mn, LDH‐Fe, and LDH‐Co after 4 and 14 days of immersion. Reproduced with permission.^[^ ^147^ ^]^ Copyright 2022, John Wiley and Sons. PVA, poly(vinyl alcohol); PAM, polyacrylamide; CS, chitosan/silk fibroin; PEO, plasma electrolytic oxidation; EIS, electrochemical impedance spectroscopy.

Mechanical properties are essential properties for materials applied in bone tissue engineering.^[^ ^102^, ^131^ ^]^ Materials with similar mechanical properties to natural bone can provide necessary support and avoid stress shielding after transplantation.^[^ ^104^, ^135^ ^]^ It is reported that pristine LDHs (Mg‐Al, Mg‐Fe, Zn‐Al, and Zn‐Fe) possess an elastic modulus of 9–35 GPa, which is similar to cortical bone (5–23 GPa).^[^ ^54^ ^]^ Most LDHs can maintain their shape and biomechanical properties after being immersed in the culture medium for a long period.^[^ ^54^, ^136^ ^]^ Besides favorable intrinsic mechanical properties, the capacity to exchange anions and incorporate long‐chain organic anions of LDHs make them one of the most used reinforcing fillers for bone tissue engineering materials.^[^ ^137^ ^]^ The composites composed of LDHs and synthetic polymer have been proved to possess favorable mechanical properties. For instance, a bioinspired multilayered film composed of LDHs and poly(vinyl alcohol) (PVA) with high tensile strength and ductility was fabricated by spin coating PVA on LDH nanosheets (**Figure** **4**a). The film shows better mechanical performance with the LDHs content increasing and possesses a tensile strength of 169.36 MPa and a lamellar bone‐comparable elastic modulus when LDH ingredient accounts for 98 wt% of the composite.^[^ ^138^ ^]^ Besides, LDH/polyacrylamide (PAM) hydrogels show perfect deformability and extraordinary stretchability, proposing a promising application in repairing intervertebral discs and muscles (Figure 4b).^[^ ^139^ ^]^ Moreover, LDHs were also reported to enhance the tensile strength and elongation of PCL scaffold.^[^ ^140^ ^]^ Despite natural polymers with high water content bear low mechanical properties, composites composed of LDH and natural polymers show enhanced mechanical properties. For example, LDH/gelatin composite fabricated with coprecipitation and solvent‐casting methods possesses Young's modulus of 19.8 ± 0.41 and 12.5 ± 0.35 GPa.^[^ ^141^ ^]^ In addition, Lv et al.^[^ ^117^ ^]^ combined MgFe‐LDH with CS hydrogel to strengthen the mechanical properties of CS hydrogel. Significantly increased storage modulus (*G*′) was observed in hydrogels with higher LDH concentrations and the 0.1 wt/vol% MgFe‐LDH/CS sample showed the best *G*. The compression mechanical properties of the MgFe‐LDH/CS hydrogels were also tested, which demonstrated that MgFe‐LDH obviously increases the compression mechanical properties of the CS hydrogel. Notably, the compression modulus of the 0.1 wt/vol% MgFe‐LDH/CS sample increases by 10.2‐fold as compared to that of the CS sample (Figure 4c).

Biodegradable materials can avoid re‐surgery to remove implant, thus reducing the physical and financial burdens on patients. However, the long duration of bone healing and remodeling (commonly taking 3–6 months) requires a slow degradation rate and high corrosion resistance of the materials.^[^ ^142^, ^143^, ^144^ ^]^ LDHs with favorable corrosion resistance have been served as coating materials for pure Mg. Higher corrosion potential, larger impedance, and less hydrogen evolution were observed in Mg‐Al LDH‐coated Mg compared with pure Mg and Mg (OH)_2_‐coated Mg (Figure 4d). The authors ascribed the enhanced corrosion resistance of Mg‐Al LDH‐coated Mg to its anion exchangeability reducing Cl^−^ attack.^[^ ^34^ ^]^ Peng et al.^[^ ^145^, ^146^ ^]^ fabricated a plasma electrolytic oxidation/MgAl‐LDH (PEO/MgAl‐LDH) composite coating to further enhance the corrosion resistance of magnesium alloy (AZ31) (Figure 4e). Reduced hydrogen evolution and corrosion rate were observed in PEO/MgAl‐LDH group compared with AZ31, PEO, and LDH groups (Figure 4f). Furthermore, when a low Zn content (1.17 at%) was added to the Mg‐Al LDH (Mg‐Zn‐Al LDH), the coating composite (PEO/Mg‐Zn‐Al LDH) acquires better corrosion resistance. In another study, Mn‐based LDH was identified to possess better corrosion resistance in comparison with Fe‐based LDH and Co‐based LDH (Figure 4g).^[^ ^147^ ^]^

**Figure 5:**
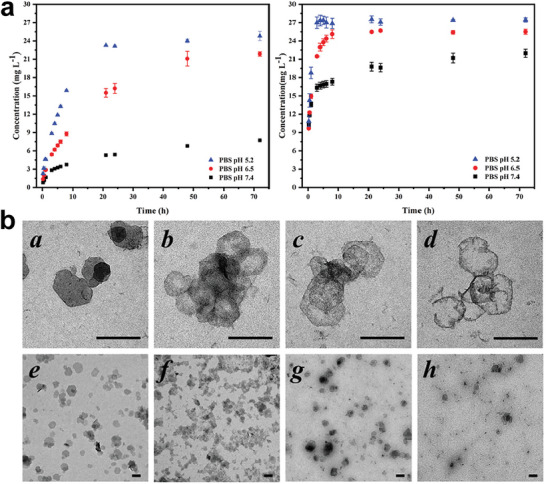
a) Degradation behaviors of larger MgAl‐LDH (left) and smaller MgAl‐LDH (right) in PBS of different pH. Reproduced with permission.^[^ ^149^ ^]^ Copyright 2023, Wiley–VCH. b) Degradation performance of FeAl‐LDH nanosheets in a buffer of pH 5.0 for a,e) 10 min, b,f) 30 min, c,g) 60 min, and d,h) 2 h; scale bar = 200 nm. Reproduced under the terms of the Creative Commons CC–BY license.^[^ ^90^ ^]^ Copyright 2018, The Authors. Published by Wiley–VCH.

The released metal cations and interlayer anions depending on the degradation behaviors contribute to the bioactivity of LDHs. As an alkaline 2D nanomaterial, LDHs possess acid‐responsive degradation properties and tend to degrade faster in acidic environments, which benefits targeted drug delivery as acidic or H_2_O_2_‐riched microenvironment is more commonly observed in bone infections, bone tumors, and osteoarthritis circumstances.^[^ ^25^, ^81^ ^]^ Of note, LDHs, composed of different metal ions, response differ with acidic environments, such as MgAl‐LDH tend to degrade much faster than ZnAl‐LDHs in acidic buffer solution.^[^ ^30^, ^148^ ^]^ The particle size can also influence the acid‐responsive biodegradability of LDHs. For instance, the larger MgAl‐LDHs nanosheets (average size 246 ± 3 nm) were proved to be more sensitive to acidic environments than the smaller MgAl‐LDHs nanosheets (average size 55 ± 2 nm) (**Figure** **5**a).^[^ ^149^ ^]^ Despite no study has systematically evaluated the degradation paradigms of various LDHs, Cao et al.^[^ ^90^ ^]^ visually recorded the degradation process of FeAl‐LDH using real‐time TEM, which demonstrated the initial decomposition of the nanosheet edge in an initial 30 min followed by a collapse of the major structure in 2 h. Meanwhile, amount H^+^ ions penetrated the interlayer gallery of the FeAl‐LDHand protonate the central OH^−^ groups, thus accelerating nanosheet collapse was observed, indicating the impact of an acidic environment on the degradation behaviors of LDHs (Figure 5b). In addition, the degradation products of LDHs can be easily metabolized and excreted through the liver and kidney, as detected in feces and urine, promising the high biocompatibility of LDHs.^[^ ^150^, ^151^ ^]^ Nevertheless, the comprehensive metabolic pathways, biodistribution, and degradation mechanisms of different LDHs need further exploration.

### 4.2 The Biocompatibility and Cell‐Responsive Properties of LDHs

The great biocompatibility of LDHs has been widely verified as studies reported the magnesium, zinc, and aluminum released from LDHs show no cytotoxic effects but promote the viability, adhesion, migration, and proliferation of co‐cultured cells.^[^ ^136^, ^140^ ^]^ Even so, the increased pH value and metal concentration in the cell culture medium were observed when co‐culturing cells with LDHs.^[^ ^23^ ^]^ As metal elements always bear some degree of biological toxicity in high concentration, a safe metal concentration range that exerts optimum therapeutic effects but does not cause cytotoxicity should be identified, especially when the traditional MgAl elements of LDHs were exchanged with other metals, since rare metal ions tend to impose a negative impact on cellular homeostasis.^[^ ^152^, ^153^, ^154^, ^155^ ^]^ It is demonstrated that aluminum is one of the less toxic metals (Na < Cr < Mg < Mo < Al < Ta < Co < Ni < Fe < Cu < Mn < V).^[^ ^156^ ^]^ Zn‐containing LDHs were reported to show higher cytotoxicity than Mg‐containing LDHs both in the form of suspension and extract.^[^ ^54^ ^]^ However, in another study conducted by Wang et al.,^[^ ^157^ ^]^ the cell viability in response to Zn‐Al LDH was reported to be 20% higher than control medium and 40% higher than MgAl‐LDH‐containing medium. Therefore, the cytotoxicity and secure concentration range of LDHs with different metal ions are not yet conclusive and further studies are needed.

**Figure 6:**
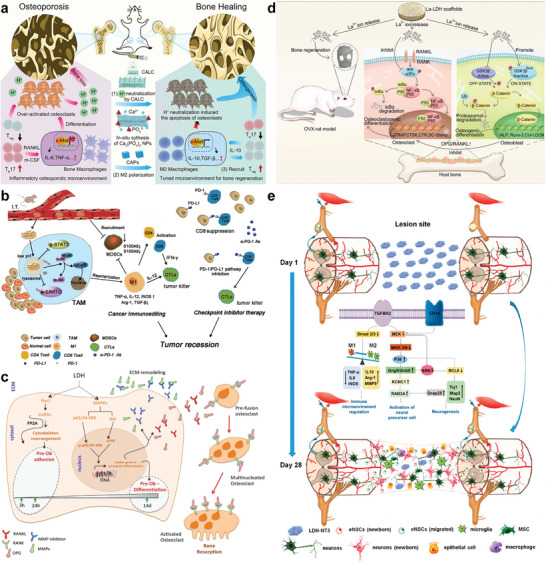
a) The regulation of the osteoporosis microenvironment by CaAl‐LDH. CaAl‐LDH liberates Ca^2+^ from osteoclast by neutralizing H^+^. Ca^2+^ mineralizes to produce calcium phosphate nanoparticles and encourage bone macrophages to polarize to M2 phenotype through activating c‐Maf transcriptional factor followed. Then anti‐inflammatory M2 macrophages secrete a lot of cytokines to modulate the bone immune microenvironment and encourage the production of new bone. Reproduced with permission.^[^ ^158^ ^]^ Copyright 2022, American Chemical Society. b) MiR155‐loaded LDH promotes M1 polarization of macrophages and enhances cellular immunosuppressive effect in the tumor environment. Reproduced under the terms of the Creative Commons CC–BY license.^[^ ^278^ ^]^ Copyright 2019, The Authors. Published by Wiley–. c) Schematic representation of the LDH‐based intracellular signaling. Reproduced with permission.^[^ ^39^ ^]^ Copyright 2018, John Wiley and Sons. d) Schematics of osteogenic effect of La‐LDH scaffolds. The composites encourage osteogenesis of rBMSCs by activating Wnt/*β*‐catenin pathway and constrain osteoclastogenesis by inhibiting NF‐*κ*B signaling pathway simultaneously. Reproduced with permission.^[^ ^35^ ^]^ Copyright 2021, Chu et al. e) LDH‐NT3 promotes neuro nactivation and nerval regeneration by modulating the immune microenvironment. Reproduced with permission.^[^ ^163^ ^]^ Copyright 2021, American Chemical Society.

Different signal pathways were involved in the biological responses of cells to LDHs nanosheet, which varies with the alteration of metal ion composition in LDHs and cell types. For example, when co‐cultured with macrophage cells, MgAl‐LDH and CaAl‐LDH can induce macrophage to polarize to M2 phenotype (anti‐inflammatory) thus promoting osteogenesis of bone defects and osteoporosis (**Figure** **6**a).^[^ ^34^ ^]^ By contrast, MiR155‐loaded MgAl‐LDH was reported to downregulate the expression level of phosphorylated STAT3 and ERK1/2 and upregulate NF‐*κ*B expression thus promoting M1 polarization of tumor‐associated macrophages (Figure 6b).^[^ ^158^ ^]^ Wu et al.^[^ ^159^ ^]^ evaluated effects of MgAl‐LDH nanoparticles on mouse embryonic stem cells (mESCs) and found MgAl‐LDH nanomaterials can maintain the self‐renewal properties and inhibit spontaneous differentiation of mESCs through upregulating pluripotent genes and downregulating lineage‐specific genes of PI3K signaling pathway. In another study, enhanced expression of pluripotency‐related genes and maintained stemness of mESCs were induced by MgFe‐LDHs, where LIFR/PI3K/AKT and LIFR/JAK/STAT3 signaling pathways were reported to be activated by MgFe‐LDHs and result in downstream p‐STAT3 activation and TET1/2 expression.^[^ ^160^ ^]^ Moreover, the upregulation of Wnt/*β*‐catenin pathway, MAPK‐dependent pathway as well as downregulation of NF‐*κ*B signaling pathway leading to enhanced osteoblastogenesis and inhibited osteoclastogenesis were reported to contribute to the potent osteogenesis properties of LDHs nanosheets (Figure 6c,d).^[^ ^35^, ^36^, ^39^, ^118^, ^161^, ^162^ ^]^ Meanwhile, Zhu et al.^[^ ^133^ ^]^ found MgAl‐LDH could induce migration, neural differentiation, L‐Ca^2+^channel activation, and inducible action potential generation of neural stem cells (NSCs) by activating transforming growth factor‐*β* receptor 2 (TGFBR2) (Figure 6e). Fernandes et al. demonstrated that cytoskeleton rearrangement, extracellular matrix (ECM) remodeling, and inflammatory landscape may account for the LDHs‐induced adhesion, proliferation, and migration of fibroblasts, among which many bioactive factors and signal pathways were mobilized.^[^ ^136^, ^164^ ^]^ In the process of activating dendritic cells, LDHs dose‐dependently induces nuclear NF‐kB expression and decreases IkBa level.^[^ ^165^ ^]^

### 4.3 LDHs for Osteogenesis

#### 4.3.1 The Intrinsic Osteogenesis Properties of LDHs

**Figure 7:**
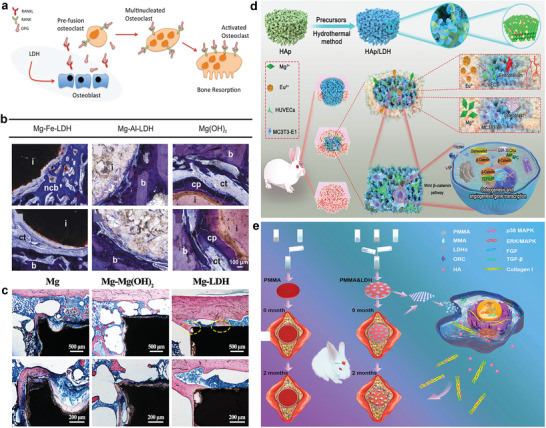
a) MgAl‐LDH and ZnAl‐LDH induce RANKL and OPG proteins expression by osteoblast that related with osteoclastogenesis. Reproduced with permission.^[^ ^39^ ^]^ Copyright 2018, John Wiley and Sons. b) Mg‐Fe‐LDH sample is surrounded by a thick layer of connective tissue and non‐calcified bone is detected at the implant interface. MgAl‐LDHs sample is in sparsely direct contact with bone while distinct fibrous capsules and corrosion products are visible around the Mg(OH)_2_ sample. Reproduced with permission.^[^ ^166^ ^]^ Copyright 2016, Elsevier. c) Histology‐stained image of the bone‐Mg, Mg‐Mg(OH)_2_ and Mg‐LDH interfaces for 8 weeks. Mg and Mg‐Mg(OH)_2_ implants are not attached to the formed bone while the newly formed bone is tightly attached to the implant in Mg‐LDH group. Reproduced with permission.^[^ ^34^ ^]^ Copyright 2021, Cheng et al. d) Schematic of in situ growth of MAE‐LDH on a porous hydroxyapatite scaffold, which enables efficient bone repair and vascular regeneration by activating the Wnt/*β*‐catenin signaling pathway. Reproduced with permission.^[^ ^118^ ^]^ Copyright 2022, Wi. e) Schematic presentation of PMMA and MgAl‐LDH microsheet‐modified PMMA bone cement, and four key osteogenic pathways in rabbit skull defect models. Reproduced with permission.^[^ ^36^ ^]^ Copyright 2021, American Chemical Society. MAE, MgAlEu; HA, hydroxyapatite; PMMA, poly(methyl methacrylate).

Given the flexibility of coupling various metals that possess osteogenic capabilities (Mg^2+^, Sr^2+^, Ca^3+^, etc.), LDHs have been widely explored in promoting bone regeneration in treating bone defects. Kang et al.^[^ ^39^ ^]^ found both MgAl‐LDH and ZnAl‐LDH can stimulate pre‐osteoblast to express osteogenic gene markers in a mitogen‐activated protein kinase (MAPK)‐dependent manner, where the differentiated osteoblasts also express increased osteogenic genes and promote collagen deposition and mineralization. Notably, MgAl‐LDHs and ZnAl‐LDHs also play an essential role in osteoblast‐induced osteoclastogenesis, proposing a therapeutic solution for modulating bone remodeling (**Figure** **7**a). For preclinical practice, LDHs as 2D materials are often combined with 3D scaffold to enhance the biocompatibility and bioactivity of pristine scaffold materials, given current orthopedic prostheses are commonly made up of bioinert stainless steel, titanium, and ceramics. Weizbauer et al. compared the osteogenic potential of MgAl‐LDH, MgFe‐LDH, and Mg(OH)_2_ as a coating material on an orthopedic prosthesis. Significantly enhanced cell proliferation was observed in MgAl‐LDH culture medium compared with MgFe‐LDH or Mg(OH)_2_ culture medium. Besides, histological results showed sparse direct bone‐implant contact in MgAl‐LDHs group, with a thick layer of connective tissue around the implant in MgFe‐LDHs and Mg(OH)_2_ group, demonstrating a superior osteoinduction and osteoconduction performance of MgAl‐LDH (Figure 7b).^[^ ^166^ ^]^ Similarly, Cheng et al.^[^ ^34^ ^]^ coated MgAl‐LDH on pure Mg and found increased osteogenic and angiogenic gene expression in vitro and enhanced bone regeneration and better osteointegration in vivo in MgAl‐LDH group compared with pure Mg (Figure 7c). More importantly, MgAl‐LDH‐coated Mg could induce macrophage to polarize to M2 phenotype (anti‐inflammatory), providing a suitable immune microenvironment for new bone formation. Reportedly, MgFe‐LDH as a modified film on titanium prosthesis was reported to generate an alkaline microenvironment that is beneficial to osteogenic differentiation and the microenvironment PH around the MgFe‐LDHs‐coated titanium is controllable though changing the Mg/Fe ratio of LDHs.^[^ ^167^ ^]^ Wang et al.^[^ ^118^ ^]^ reported the surface modification of MgAlEu‐LDH (MAE‐LDH) nanosheets on microporous hydroxyapatite (HA) scaffolds as an effective strategy to significantly enhance the cell adhesion properties of pristine HA scaffolds by improving surface roughness and hydrophilicity of HA scaffolds (Figure 6d). In vitro and in vivo experiments verified the sustained released Mg^2+^ from MAE‐LDH can promote bone regeneration and remolding. The transcriptome sequencing analysis reveals the Wnt/*β*‐catenin signaling pathway was involved in the osteogenic process of HA/MAE‐LDH scaffolds. Eskandari et al.^[^ ^168^ ^]^ prepared a novel *β*‐tricalcium phosphate‐based nanocomposite containing different concentrations of MgAl‐LDH (*β*‐TCP‐LDH) with favorable biocompatibility, porosity, and mechanical property. The optimal concentration of LDH is 10 wt% with 77% porosity for enhanced cell adhesion and 231.4 MPa compressive modulus for supplying sufficient support, proposing a promising alternative therapy for large bone defect treatment.

Natural polymer is a class of materials composed of molecules derived from natural compounds linked by covalent bonds and has been extensively explored in bone tissue engineering. LDHs have also been combined with natural polymers to promote their osteogenic and biomechanical performance. For example, a novel CaAl‐LDH‐HA/gelatin scaffold with mechanical properties between spongy bone and cortical bone was fabricated using coprecipitation and solvent‐casting methods. The scaffold possesses outstanding osteogenic properties and favorable biocompatibility inducing negligible immunoreaction after implantation. Significant new bone formation and accelerated bone defect reunion were observed in the rabbit radius after scaffold implantation. Besides, vitamin D3 (VD3) has been encapsulated within the gelatin to further improved the biomineralization and cellular response properties of the scaffold.^[^ ^169^ ^]^ Moreover, the osteogenic properties of CaAl‐LDH‐HA/gelatin scaffold can be further strengthened by adipose tissue‐derived stromal cells (ADSC) co‐implantation, as observed in histomorphometric findings.^[^ ^141^ ^]^ Cao et al.^[^ ^170^ ^]^ combined ternary MgSrFe‐LDH with chitosan to fabricate a composite scaffold with potent osteogenic properties. The osteogenesis of the composite scaffold was considered to be attributed to the synergistic effect of the sustained released Mg^2+^, Sr^2+^, and Fe^3+^. Besides, the MgSrFe‐LDH/chitosan scaffold presents a 3D interconnected porous structure with a pore size ranging from 100 to 300 µm, which is also in favor of bone ingrowth and osteointegration.

Synthetic polymers are also extensively modified with LDHs to improve biocompatibility and osteogenic properties. Baradaran et al.^[^ ^171^ ^]^ reported that the poly(e‐caprolactone)/MgAl‐LDH (PCL/LDH) nanocomposite scaffolds possess good competence in protein adsorption and mechanical properties to remarkedly facilitate attachment and proliferation of co‐cultured cells. Moreover, in vitro cell culture studies showed the alkaline phosphatase (ALP) activity of PCL/LDH samples was conspicuously increased, demonstrating the fabricated microsphere‐aggregated scaffold is capable of inducing osteogenic differentiation of mesenchymal stem cells (MSCs). Zhou et al.^[^ ^172^ ^]^ fabricated a polymer network (PN) composed of layered poly(lactide‐*co*‐caprolactone) copolymer and MgAl‐LDH, the PN can continuously release magnesium ions and promote the osteogenic gene expression of co‐cultured cells. Wang et al.^[^ ^173^ ^]^ combined MgAl‐LDH with poly(*N*‐isopropylacrylamide) (PNIPA) and nano‐HA to synthesize a thermosensitive composite (PNIPA‐LDH‐nHA). The PNIPA‐LDH‐nHA scaffold was proved to possess favorable biocompatibility, incredible mechanical toughness, and outstanding osteogenic properties. Poly(ethylene glycol) (PEG) crosslinked by polydopamine‐coated MgAl‐LDH (PD‐LDH) presenting robust bioactivity and excellent adhesion properties was also designed.^[^ ^174^ ^]^ Enhanced osteogenic differentiation of human mesenchymal stem cells (hMSCs) was observed in the composite medium. For total joint arthroplasty, poly(methyl methacrylate) (PMMA)‐based bone cement combining MgAl‐LDH nanocomposites with enhanced osteogenic properties was fabricated. On gradually substituting Mg with Al in LDH, the nanocomposites showed increasing fatigue behavior and osteogenesis property verified by in vitro and in vivo studies in rabbits.^[^ ^175^, ^176^ ^]^ The PMMA/MgAl‐LDH nanocomposites with excellent osteogenic properties were also fabricated by Wang et al.,^[^ ^36^ ^]^ where the nanocomposites can induce 18.34‐fold new bone formation in rabbit skull defect models compared with pristine PMMA (Figure 7e). Moreover, the maximum polymerization reaction temperature of PMMA/MgAl‐LDH decreased by 7.0 °C compared with that of pristine PMMA, alleviating the negative effects of high temperature on surrounding cells. The biomechanical properties of PMMA/MgAl‐LDH also decreased slightly thus reducing stress‐shielding osteolysis, thus promoting osseointegration.

#### 4.3.2 LDHs Loaded with Osteogenic Drugs and Bioactive Factors

**Figure 8:**
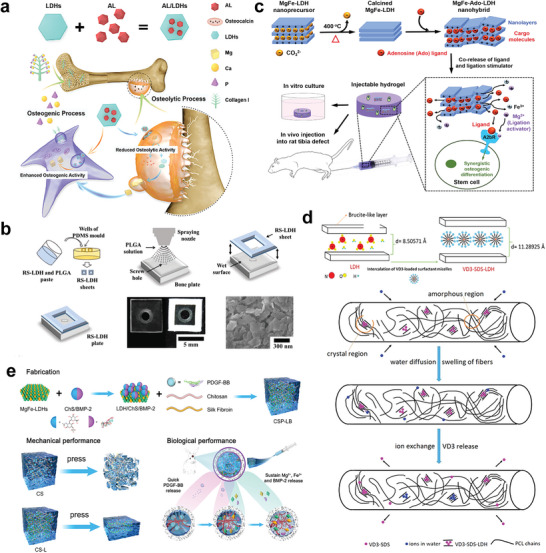
a) Diagrammatic illustration of the excellent osteogenic differentiation and bone regeneration ability of MgAlYb‐LDH/AL composite, which achieves an increase in bone density by enhancing osteogenic activity and inhibiting osteolytic activity. Reproduced with permission.^[^ ^32^ ^]^ Copyright 2020, Royal Society of Chemistry. b) Schematic drawing of manufacturing and functional testing of RS‐LDH‐PLGA. RS‐LDH‐PLGA is formulated on top of an unmodified bioresorbable bone plate. Reproduced with permission.^[^ ^178^ ^]^ Copyright 2016, John Wiley and Sons. c) Schematic diagram of the preparation of MgFe‐Ado‐LDH nanohybrid and their synergistic induction of osteogenic differentiation of stem cells in vitro and in vivo. Reproduced with permission.^[^ ^179^ ^]^ Copyright 2017, Elsevier. d) Diagrammatic presentation of an LDH/PCL electrospun holder loaded with vitamin D3 and the release process of vitamin D3 from nanohybrid‐enriched PCL fibers. Reproduced with permission.^[^ ^180^ ^]^ Copyright 2020, Elsevier. e) Schematics of the preparation of MgFe‐LDH‐modified CS hydrogel with enhanced mechanical and biological properties. Reproduced with permission.^[^ ^117^ ^]^ Copyright 2022, John Wiley and Sons. AL, alendronate; RS, risedronate; PLGA, poly(lactide‐*co*‐glycolic acid); FE‐SEM, field‐emission scanning electron microscopy; Ado, adenosine.

With excellent drug loading and sustained delivery abilities, LDHs have been applied as osteogenic drugs and bioactive factors loading and delivery carriers. Given the intrinsic osteogenic properties of LDHs, drugs or bioactive factors‐loaded LDHs strategies are expected to present more excellent osteogenic performance. Wang et al.^[^ ^32^ ^]^ found Yb‐containing MgAl‐LDH monolayer nanosheets (MgAlYb‐LDH) have a loading content and encapsulation efficiency of 97% and 98% for alendronate (AL, a bone resorption inhibitor and used to treat osteoporosis). The MgAlYb‐LDH/AL composites were proved to possess an outstanding osteogenic differentiation and bone regeneration ability, which induce 1.41‐fold new bone formation in the osteonecrosis of the femoral head model of rabbit compare with a positive control group (autologous bone graft, clinical gold standard) at 8 weeks postoperatively (**Figure** **8**a). Another similar study intercalated AL into MgAl‐LDH and ZnAl‐LDH forming a novel nanohybrid (AL‐LDH) to realize continuous and enhanced AL release. 10.6‐fold higher accumulated AL content in MG63 cells was observed in the AL‐LDH group compared with AL group after 1‐h incubation, therefore promoting the in vitro proliferation level and ALP activity of MG63.^[^ ^177^ ^]^ Another osteogenic drug called risedronate (RS) was also loaded in LDHs to improve the osteogenic properties of the LDHs/poly(lactide‐*co*‐glycolic acid) (PLGA) scaffold.^[^ ^178^ ^]^ Sustained drug release and expedited bone regeneration were achieved in the RS‐LDHs‐PLGA group compared with the LDHs‐PLGA group (Figure 8b).^[^ ^178^ ^]^ Adenosine (Ado) is an endogenous nucleoside that exerts various physiological functions in different biological processes. Kang et al.^[^ ^179^ ^]^ intercalated Ado into the interlayer space of MgFe‐LDH to realize the co‐deliver of Ado and Mg^2+^ ions, the Ado as a ligand and Mg^2+^ ion as a ligation activator can synergistically activate A2bR, which significantly promotes osteogenic differentiation of stem cells and new bone formation of defective tibial bone compared with that of unmodified Ado (Figure 8c). Besides, a VD3‐loaded LDH/PCL electrospun scaffold was synthesized by Belgheisi et al. (Figure 8d).^[^ ^180^ ^]^ The released VD3 was found to support cell adhesion and proliferation as well as facilitate apatite‐like crystal formation and new bone formation.

On the other hand, LDHs have also been used to load and deliver osteogenic bioactive factors to promote endogenous bone regeneration. Lv et al.^[^ ^117^ ^]^ incorporated bone morphogenetic protein 2 (BMP‐2)‐modified MgFe‐LDH nanosheets into platelet‐derived growth factor‐BB (PDGF‐BB)‐loaded chitosan/silk fibroin (CS) to fabricate a smart thermosensitive hydrogel (CSP‐LB). The sustained released BMP‐2 and Mg^2+^/Fe^3+^ ions endow the scaffold with potent osteogenic properties with 16.2‐fold new bone formation was observed in the CSP‐LB group compared with CS group. Moreover, the MgFe‐LDH‐modified CS hydrogel presents shorter gelation time, lower sol–gel transition temperature, but enhanced mechanical properties that are more favorable for clinical practice (Figure 8e). Chen et al.^[^ ^181^, ^182^ ^]^ designed and fabricated a PFT*α*‐loaded LDH‐chitosan porous scaffold (LDH‐CS‐PFT*α*) that possesses great PFT*α* delivery ability and outstanding osteogenic properties. The 3D interconnected macropores of the scaffold can facilitate human bone marrow stem cell (hBMSCs) adhesion and bone ingrowth. Better stem cell osteogenic differentiation in vitro and enhanced bone regeneration volume in vivo were also observed in LDH‐CS‐PFT*α* group compared with the LDH‐CS group, which was ascribed to the potent osteogenic characteristics of PFT*α*.

### 4.4 LDHs for Angiogenesis

**Figure 9:**
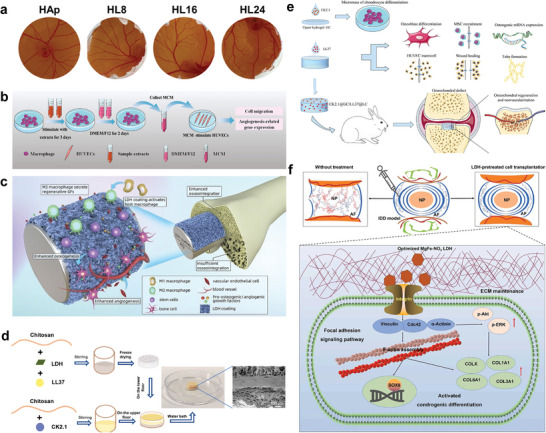
a) Gross photographs of neovascularization induced by HAp and scaffolds with different hydrothermal treatment times (8, 16, and 24 h) of the chicken chorioallantoic membrane assay. Reproduced under the terms of the Creative Commons CC–BY license.^[^ ^118^ ^]^ Copyright 2022, The Authors. Published by Wiley–VCH. b) Macrophages cultured with MgAl‐LDH‐coated Mg extract secrete angiogenic factors that promote cell migration and angiogenesis‐related gene expression. Reproduced with permission.^[^ ^34^ ^]^ Copyright 2021, Elsevier. c) Mg‐Al LDH film promotes bone differentiation and angiogenesis. Reproduced with permission.^[^ ^34^ ^]^ Copyright 2021, Elsevier. d) Synthetic schematic representation of CK2.1@GC/LL37@LC scaffold. The scaffold shows good two‐phase connection, which overcome the problem of difficult combination of dual‐phase materials. Reproduced with permission.^[^ ^192^ ^]^ Copyright 2021, Elsevier. e) CK2.1@GC/LL37@LC scaffold is favorable for osteochondral regeneration and neovascularization. Reproduced with permission.^[^ ^192^ ^]^ Copyright 2021, Elsevier. f) Schematics of the function and mechanism of IDD model with or without LDH treatment. Without LDH treatment, the intervertebral disc was characterized of reduced disc height, destroyed structure, and loss of physiological function. While, the optimized LDH‐pretreated cell transplantation enhanced the chondrogenic differentiation and in situ intervertebral disc regeneration through focal adhesion signaling pathways. Reproduced with permission.^[^ ^200^ ^]^ Copyright 2023, Elsevier. HAp, hydroxyapatite; CK2.1@GC, CK2.1 coated *β*‐glycerophosphate/chitosan; LL37@LC, LL37‐modified MgAl‐LDH/chitosan; IDD, intervertebral disc degeneration.

As the main source of nutrients such as oxygen, serum protein, and growth factors used for supporting bone regeneration are supplied by vascular system, the angiogenesis properties are essential for materials applied in bone tissue engineering to reconstruct microcirculation network thus promoting bone remodeling.^[^ ^183^, ^184^, ^185^, ^186^ ^]^ As many metal ions possess great angiogenesis characteristics (Cu^2+^, Eu^3+^, Mn^2+^, Ni^2+^, etc.) while others with osteogenesis properties (Mg^2+^, Ca^2+^, Fe^3+^, etc.), LDH‐based tissue engineering scaffolds with multiple metal doping and release abilities hold the potential to couple both angiogenesis and osteogenesis properties.^[^ ^62^, ^187^, ^188^, ^189^ ^]^ For example, Wang et al.^[^ ^118^ ^]^ functionalized MgAlEu‐LDH nanosheets on microporous HA to fabricate a scaffold with outstanding osteogenesis and angiogenesis properties. The Eu^3+^ sustained released from MgAlEu‐LDH effectively contributes to vascular regeneration as verified by in vitro studies including scratch assay, transwell assay, and tube formation assay. Angiogenic gene markers including Wnt1 and VEGF were upregulated in MgAlEu‐LDH/HA group in comparison with HA group. In vivo study investigating the CD31 (a neovascular marker) expression found significantly higher CD31 fluorescence intensity in MgAlEu‐LDH/HA group compared with HA group. Moreover, higher CD31 fluorescence intensity was observed in scaffolds with higher LDHs content. More neovascularization was also observed in MgAlEu‐LDH/HA group compared with HA group revealed by the chicken chorioallantoic membrane assay, suggesting the excellent in vivo angiogenesis property of MAE‐LDH (**Figure** **9**a). Zhang et al.^[^ ^147^ ^]^ reported that the Mn^2+^ and Fe^3+^ ions released from MnFe‐LDH propose a positive influence on the angiogenesis of human umbilical vein endothelial cells (HUVECs). The Ni^2+^ ions released from NiTi‐LDH were also proved to support the angiogenesis behavior of HUVECs.^[^ ^190^ ^]^ In addition to coupling rare angiogenic metal ions, the regular MgAl‐LDH and MgFe‐LDH were also proven to possess favorable angiogenic properties. For example, Cheng et al.^[^ ^34^ ^]^ reported that the macrophages cultured with MgAl‐LDH‐coated Mg extract can secrete angiogenic factors that promote cell migration, angiogenic gene expression, and neovascular formation of HUVECs (Figure 9b,c). A slight evaluation of local pH value and Mg^2+^ concentration was considered to contribute to the angiogenic microenvironment. Similar results are obtained in another study where MgAl‐LDH was modified on Mg alloy AZ31 to enhance its angiogenesis properties.^[^ ^191^ ^]^ Lv et al.^[^ ^117^ ^]^ combined MgFe‐LDH nanosheets with chitosan/silk fibroin (CS) hydrogel and found the MgFe‐LDH/CS hydrogel showed significantly enhanced cell migration and tube formation properties than pristine CS hydrogel, indicating the great angiogenic properties of MgFe‐LDH nanosheets.

In addition, angiogenic bioactive molecules have been also combined with LDHs to further enhance the angiogenesis properties of LDH‐based nanomaterials. Liu et al.^[^ ^192^ ^]^ manufactured a bilayer peptide‐loaded composite incorporating upper CK2.1‐coated *β*‐glycerophosphate/chitosan (CK2.1@GC) for cartilage repair and lower LL37‐modified MgAl‐LDH/chitosan (LL37@LC) for vascularized bone regeneration, of which LL37 is a cathelicidin peptide that can promote neovascularization by inducing the proliferation and migration of epithelial cells (Figure 9d,e). Collectively, in vitro results found enhanced HUVECs migration and more tube formation in LL37@LC group compared to LC group, indicating the favorable angiogenesis properties of LL37@LC.

### 4.5 LDHs for Chondrogenesis

The degenerative change of articular cartilage are typical pathophysiologic characteristics of osteoarthritis (OA).^[^ ^4^, ^193^, ^194^, ^195^ ^]^ Tissue engineering of osteochondral tissue, bone–cartilage interface defect, and intervertebral disc also requires the chondrogenesis properties of involved biomaterials.^[^ ^196^, ^197^, ^198^, ^199^ ^]^ LDHs with inherent chondrogenesis properties and excellent chondrogenic drugs or bioactive factors loading and delivery abilities hold the potential to repair chondral defects and halt the progression of OA. Reasonably, studies have paid close attention to the role of LDHs in promoting chondrogenesis. Wang et al.^[^ ^200^ ^]^ synthesized and optimized the ion elemental compositions of Mg‐based LDHs to induce chondrogenic differentiation of human umbilical cord mesenchymal stem cells (hUCMSCs). With the co‐culture time of hUCMSCs and Mg‐based LDHs extended, more glycosaminoglycan (GAG) deposition was observed by Alcian blue staining. qPCR and immunofluorescence analyses also revealed the enhanced chondrogenic differentiation marker expression of Mg‐based LDH‐treated hUCMSCs. Then, intervertebral disc degeneration (IDD) model in rats was established and transplanted with MgFe‐LDH‐treated hUCMSCs. Radiographical and immunohistochemical revealed a more complete recovery of the disc space height and integrated tissue structure in MgFe‐LDH/hUCMSCs group compared with control group and untreated hUCMSCs group, indicating the potent chondrogenic properties of MgFe‐LDH. Subsequently, transcriptome sequencing found ECM formation and cell adhesion signaling pathway were upregulated in the MgFe‐LDH‐mediated chondrogenesis processes (Figure 9f).

Besides, LDHs loaded with chondrogenic drugs and siRNA for enhanced and effective cartilage repair have also been explored. Lee et al.^[^ ^40^ ^]^ developed well‐defined micrometer‐sized trifluoroacetate (RGD)‐coated MgAl‐LDH, which were wrapped in a gel formed by thermal energy driving. Kartogenin (KGN), a small molecular compound that was reported to be effective in inducing chondrogenic differentiation of MSCs was then loaded in the RGD‐coated MgAl‐LDH. Apart from sustained release of KGN, the MgAl‐LDH was proved to improve the rigidity of ECM and enhance MSCs adhesion. Significantly, the immunofluorescence study sheds light on the augmented chondrogenic differentiation of cells in this nanocomposite system, simultaneously, the enhanced expression of type II collagen and SOX9 were also observed at both mRNA and protein levels. In addition, Yang et al.^[^ ^201^ ^]^ fabricated a novel injectable and thermoresponsive siRNA delivery hydrogel composed of poly(*N*‐isopropyl acrylamide) (pNIPAAM) and either MgAl‐LDH or MgFe‐LDH nanoplatelets with exceptional biocompatibility. Through fluorescently labeled transfection indicator, the pNIPAAM/LDH hydrogels showed superior ability in siRNA delivery as intracellular fluorescence‐labeled siRNA was significantly increased in pNIPAAM/LDH group in comparison to hyaluronan and fibrin gels and was absent in pNIPAAM hydrogel without LDH platelets, indicating outstanding intracellular siRNA delivery ability of MgAl‐LDH and MgFe‐LDH nanoplatelets, which provide a potential solution for cartilaginous degeneration by effectively downregulating the expression level of degenerative factors in chondrocytes.

## 5 LDHs for Bone Infection Treatment

**Figure 10:**
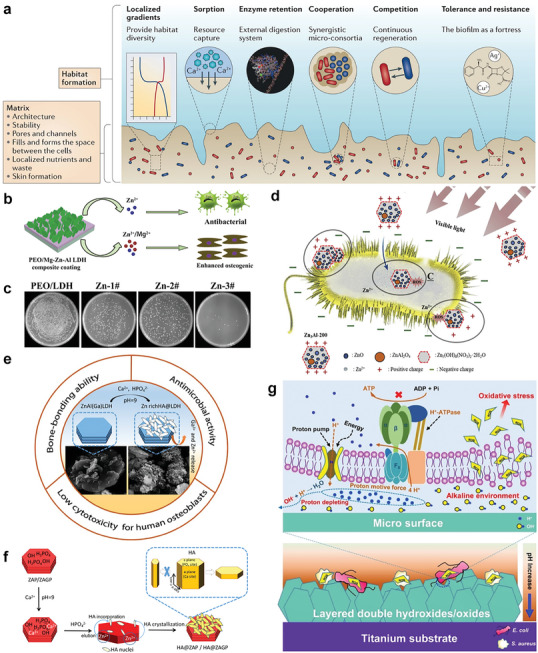
a) Schematic representation of biofilm formation, in which the matrix produced by bacteria forms the physical basis of the biofilm, providing structure and stability to the biofilm. Reproduced with permission.^[^ ^206^ ^]^ Copyright 2016, Springer Nature. b) PEO/Mg‐Zn‐Al LDH composite coating enhances antibacterial and osteogenic activity by releasing Zn^2+^ and Mg^2+^. Reproduced with permission.^[^ ^145^ ^]^ Copyright 2018, American Chemical Society. c) Photographs of *Staphylococcus aureus* colonies on agar culture plates and the higher Zn component results in the stronger antibacterial effect. Reproduced with permission.^[^ ^145^ ^]^ Copyright 2018, American Chemical Society. d) Schematic illustration describing the antibacterial effects of Zn^2+^ toward Escherichia coli. Reproduced with permission.^[219]^ Copyright 2019, Elsevier. e) Zn‐Rich LDHs hydroxyapatite possesses bone‐bonding ability, antibacterial activity, and low cytotoxicity for human osteoblasts. Reproduced with permission.^[^ ^220^ ^]^ Copyright 2021, American Chemical Society. f) Schematic diagram of the HA growth on LDH. In the presence of LDH, HA crystallizes mainly in the form of flake crystals. Reproduced with permission.^[220]^ Copyright 2021, American Chemical Society. g) Diagrammatic presentation indicating that the creation of a local alkaline microenvironment by LDH on the titanium surface inhibit bacterial growth by inducing oxidative stress and inactivating ATP synthesis. Reproduced with permission.^[^ ^217^ ^]^ Copyright 2018, American Chemical Society. PEO, plasma electrolytic oxidation; HA, hydroxyapatite.

The overwhelming majority of bone infections originate from the orthopedic prosthesis.^[^ ^5^, ^202^ ^]^ Periprosthetic infection with substantial patient morbidity and accounts for more than 25% of revisions is a catastrophic complication for orthopedic prosthesis‐implanted patients.^[^ ^5^ ^]^ Periprosthetic infections are harder to treat than common infections because biofilms formed in periprosthetic infections prevent conventional systemic antibiotics from reaching the infection sites and entering the bacteria (**Figure** **10**a).^[^ ^203^, ^204^, ^205^, ^206^ ^]^ At the same time, postoperative patients bear a poor immune barrier thus less responsive to antibiotics and are more prone to develop drug resistance.^[^ ^207^, ^208^, ^209^ ^]^ Therefore, traditional antibiotics are difficult to treat periprosthetic infections effectively.^[^ ^210^ ^]^ On the contrary, the bacteria adsorption capability of LDHs driven by electrostatic interaction makes them promising nanomaterials for targeted and efficient periprosthetic infection treatment. The advantages of LDHs for treating bone infections lie in three aspects: 1) Serious metal ions that have been proven to possess potent antibacterial ability (Zn^2+^, Mn^2+^, Cu^2+^, Ni^2+^, Co^2+^, etc.) can be incorporated into LDHs nanosheets thus endow LDHs with favorable antibacterial properties.^[^ ^211^, ^212^, ^213^, ^214^ ^]^ 2) ROS generated by specific LDHs (e.g., Zn‐LDHs and Ti‐LDHs) with outstanding photocatalytic performance and abundant hydroxyl groups on the surface also contribute to infection elimination.^[^ ^77^ ^]^ 3) The excellent antibiotics loading and delivery ability further enhance the antibacterial ability of LDHs. 4) pH‐sensitive biodegradability of LDHs makes them biodegrade faster and release more antibacterial metal ions and antibiotics in infection sites with lower‐pH microenvironments.^[^ ^215^, ^216^ ^]^ 5) An appropriate local alkaline environment caused by LDHs can repress the reproduction of both Gram‐positive and Gram‐negative bacteria taking advantage of inactivating ATP synthesis and inducing oxidative stress.^[^ ^217^, ^218^ ^]^

### 5.1 The Intrinsic Antibacterial Properties of LDHs

Different antibacterial metal ions‐based LDHs have been used for the prevention and treatment of bone infections, of which Zn^2+^, Ga^3+^, and Cu^2+^ were the most explored metal ions. Awassa et al.^[^ ^42^ ^]^ discovered that ZnAl‐LDH nanoparticles can specifically recognize and frequently cling to *Staphylococcus aureus* to effectively impede bacterial development though destroying bacterial membrane. Peng et al.^[^ ^145^ ^]^ coated PEO/MgZnAl‐LDH composites on Mg alloy implant by hydrothermal treatment to endow the implant with enhanced osteogenic properties and strong antimicrobial ability against *S. aureus* by sustained releasing antibacterial Zn^2+^ ions (Figure 10b). Significantly reduced *S. aureus* colonies were observed in PEO/MgZnAl‐LDH group and MgZnAl‐LDH with higher Zn component showed stronger antibacterial effects (Figure 10c). Li et al.^[^ ^219^ ^]^ introduced a more uniform ZnO‐dotted LDHs made hydrothermally to treat the Zn_3_Al‐LDH precursor, which increased the surface exposure, exerted a potent ROS‐associated damage toward *Escherichia coli*, and extends the antibacterial duration of LDHs up to 4 days (Figure 10d). Donnadio et al.^[^ ^220^ ^]^ chose antimicrobial Zn^2+^ and Ga^3+^ as the intralayer composition of LDH to synthesize ZnAl‐LDH and ZnAlGa‐LDH systems that can induce spontaneous HA deposition (Figure 10e). The obtained composites exhibited potent antibacterial activity against *S. aureus* and *Pseudomonas aeruginosa* in a concentration especially when Ga^3+^ was present in LDH composition (Figure 10f). Li et al.^[^ ^218^ ^]^ designed an effective protocol to prepare SrGa‐LDH film on the native titanium substrate using the hydrothermal method, which was further calcined to improve its alkalinity and stability and signed as LDH250. Colony culture results showed the colony‐forming units of *E. coli* and *S. aureus* formed on LDH250 substrates were apparently lower than that of naive Ti and LDH substrates. Structural damages of the bacteria were also observed in the LDH250 group under the scanning electron microscope (SEM). From a molecular perspective, higher ROS levels and lower ATP levels were found in LDH250 and LDH groups compared with pristine titanium substrate. The antibacterial mechanism of LDHs was explained as more OH^−^ ions in an alkaline microenvironment caused by LDHs can enter the electron transfer chain of bacteria and combine with f‐type H^+^ ATPase, thereby reducing ATP production. Additionally, the sustained release of Ga^3+^ not only increases ROS level but also interferes with Fe^3+^ metabolism, thus damaging the homeostasis of bacteria. Tan et al.^[^ ^217^ ^]^ also found the local alkaline microenvironments around material surfaces created by MgAl‐LDH can significantly inhibit the growth of both Gram‐positive and Gram‐negative bacteria by inducing oxidative stress and inactivating ATP synthesis (Figure 10g). Segura‐Pérez et al.^[^ ^221^ ^]^ reported a novel bone implant composed of HA and LDHs with different metal compositions, where significant bacteriostatic effects against clinical multi‐resistant bacteria under a very low minimum inhibitory concentration (0.5 mg mL^−1^) were observed in Cu‐based LDHs. Zhang et al.^[^ ^147^ ^]^ compared the antibacterial efficiency of Mg alloy coated with Mn‐based LDH, Fe‐based LDH, and Co‐based LDH. It turned out that Fe‐based LDH possessed the best antibacterial properties and Mn‐based LDH revealed excellent photothermal and enzymatic activities. Therefore, an optimized bilayer MnFe‐LDH was fabricated and obtained both intrinsic and photothermal antibacterial properties for effective bone infection treatment.

### 5.2 LDHs Loaded with Antibiotics

**Figure 11:**
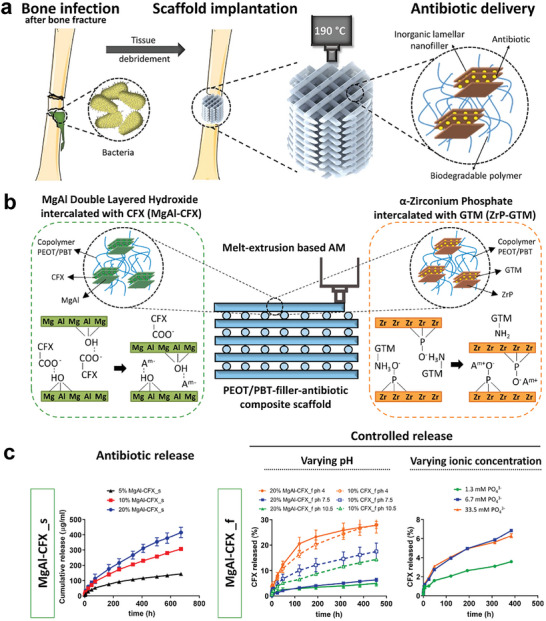
a) 3D scaffold implantation intercalated with antibiotics could prevent bone infection and promote bone regeneration during tissue reconstruction. Reproduced with permission.^[^ ^33^ ^]^ Copyright 2021, Elsevier. b) Schematic diagram of the antibacterial composite scaffolds containing either MgAl intercalated with CFX (MgAl‐CFX), or ZrP intercalated with GTM (ZrP‐GTM). The release of antibiotics confer antimicrobial activity to the scaffolds. Reproduced with permission.^[^ ^33^ ^]^ Copyright 2021, Elsevier. c) Antibiotic release profiles from MgAl‐CFX copolymer composites under different eluent conditions and influence of the ionic concentration of the eluent solution on the cumulative release percentage of CFX. Reproduced with permission.^[^ ^33^ ^]^ Copyright 2021, Elsevier. CFX, ciprofloxacin; GTM, gentamicin; dPBS, Dulbecco's phosphate‐buffered saline.

With excellent drug loading and release abilities, antibiotics delivery systems based on LDHs have been developed to combat bone infection. Chakraborti et al.^[^ ^222^ ^]^ intercalated various antibiotics including tetracycline, doxorubicin (DOX), 5‐fluorouracil, vancomycin (VAN), sodium fusidate (SF), and antisense oligonucleotides into MgAl‐LDH to realize controllable drug release. A reduced burst phase of drug release and a prolonged release time was observed in antibiotics‐LDH‐PLGA films, providing an alternative strategy for infection that need sustained antibiotics exposure. A biodegradable PLGA film loading tetracycline and AL were also intercalated in LDH nanoparticles and also achieved slow and control drug release.^[^ ^223^ ^]^ Hesse et al.^[^ ^224^ ^]^ coated a mixture of LDH compound and ciprofloxacin on Bioverit II prostheses, which was implanted into the middle ear bones of male New Zealand White rabbits infected with *P. aeruginosa*. Blood routine examination and histological analyses found no infection manifestations, indicating the outstanding abilities of LDH‐based nanomaterials as a drug delivery system for ciprofloxacin resisting. Moreover, dual‐antibiotics‐loaded LDHs were also designed and fabricated. For example, Camara‐Torres et al.^[^ ^33^ ^]^ developed an LDH‐based 3D scaffold intercalated with dual antibiotics to prevent bone infection and promote bone regeneration (**Figure** **11**a). Specifically, the biodegradable copolymer poly(ethyleneoxideterephthalate)/poly(butyleneterephthalate) (PEOT/PBT) was fabricated with melt extrusion and was combined with the ciprofloxacin‐loaded MgAl‐LDH (MgAl‐CFX) and gentamicin‐loaded *α*‐zirconium phosphates (MgAl‐CFX) (Figure 11b). The filler concentration‐dependent antibiotic release can be obtained under different eluent conditions, where the MgAl‐CFX composite presents a more sustained drug release property (Figure 11c). Besides, the incorporated antibiotics in LDH have been proved to propose neither adverse effects on the hBMSCs viability nor their osteogenic differentiation verified by matrix mineralization and osteogenic gene expression detection.

## 6 LDHs for Bone Tumor Treatment

**Figure 12:**
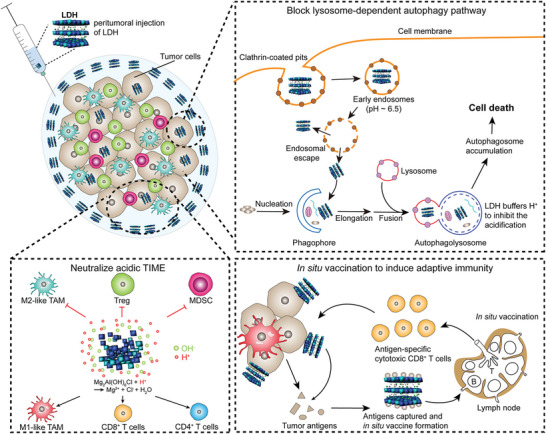
Schematic diagram of the antitumor mechanism of LDH nanoparticles. LDH nanoparticles released OH^−^ neutralize acid TIME, transforming TAMs from M2‐like to M1‐like phenotypes, increasing CD8^+^/CD4^+^ T cells while decreasing suppressive MDSCs and Tregs. LDH nanoparticles block lysosome‐dependent autophagy pathway and Tumor antigens released from the dead tumor cells were captured by LDH nanoparticles to form in situ vaccines, which then elicited an antitumor immune response of the immune system. Reproduced with permission.^[^ ^232^ ^]^ Copyright 2022, American Chemical Society. TIME, tumor immune microenvironment; TAMs, tumor‐associated macrophages; MDSCs, myeloid‐derived suppressor cells.

**Figure 13:**
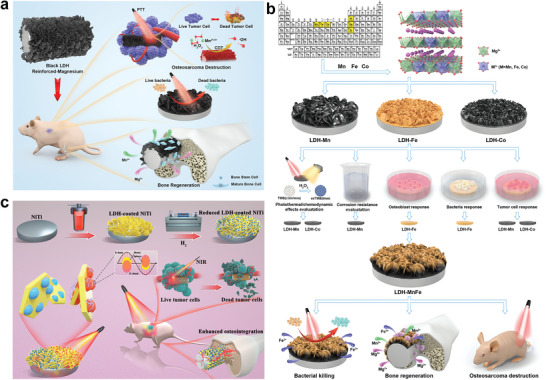
a) Schematic representation of the antibacterial, antitumor, and bone regeneration functions of black LDH reinforced‐magnesium. Reproduced with permission.^[^ ^236^ ^]^ Copyright 2022, Elsevier. b) Mn‐, Fe‐, and Co‐incorporated LDHs have versatile orthopedic application potential. Reproduced with permission.^[^ ^147^ ^]^ Copyright 2022, John Wiley and Sons. c) Diagrammatic presentation of the preparation of LDH‐H2 samples and their tumor‐killing effect in vivo. Reproduced with permission.^[^ ^190^ ^]^ Copyright 2022, American Chemical Society.

Malignant bone tumors with high malignancy seriously threaten the lives of patients and lack effective treatment.^[^ ^225^, ^226^ ^]^ Clinical strategies for bone tumors include tumor segmentectomy and postoperative chemotherapy.^[^ ^227^, ^228^ ^]^ Surgical tumor segmentectomy is usually unable to completely eliminate bone tumor cells, resulting in postoperative tumor recurrence and metastasis.^[^ ^229^ ^]^ Chemotherapies also tend to cause systematic adverse effects such as liver dysfunction, cardiotoxicity, and myelosuppression.^[^ ^230^, ^231^ ^]^ With the development of nanotechnology, innovative biomaterials for bone tumor treatment have been widely exploited, of which LDHs with fantastic physicochemical properties hold several advantages in treating bone tumor. First, LDHs as an efficient adjuvant can offer long‐term and powerful acid neutralization in the tumor microenvironment, block the lysosomal‐mediated autophagy pathway of tumor cells, and raise amounts of antitumor‐related macrophages and T cells, realizing a safe and effective bone tumor immunotherapy (**Figure** **12**).^[^ ^232^ ^]^ Second, the intrinsic photothermal and photodynamic characteristics of LDHs propose promising near‐infrared light‐based therapies, which can be further enhanced by intercalating different photosensitizer to interlayer space of LDHs.^[^ ^233^, ^234^, ^235^ ^]^ Third, positively charged LDHs with excellent drug loading capability prefer to interact with negatively charged cell membranes for effective drug delivery. Notably, LDHs with favorable photothermal characteristics have been applied to treat bone tumors. For example, Zhang et al.^[^ ^236^ ^]^ designed a black Mn‐containing LDH nanosheets‐modified Mg‐based implant, which was accompanied by a favorable photothermal effect and nanocatalysis Fenton‐like performance (**Figure** **13**a). In vitro and in vivo studies demonstrated the black LDH‐modified magnesium alloy possesses effective tumor‐killing ability. Similar results can be seen in another study conducted by Zhang et al.,^[^ ^147^ ^]^ where the corrosion resistance, osteogenic properties, antibacterial, and anti‐osteosarcoma capabilities of Mn‐, Fe‐, and Co‐incorporated LDHs were investigated, respectively. It turned out the MnFe‐LDH possess best osteogenic properties and exert a potent bacterial killing and osteosarcoma destruction effects under near‐infrared irradiation (Figure 13b). In addition, LDH‐based photodynamic therapy (PDT) has also been explored for tumor elimination. For example, Gao et al.^[^ ^89^ ^]^ reported LDH‐based near‐infrared laser‐activated supramolecular photosensitizers for highly efficient two‐photon PDT. The monolayer and interface‐restricted microenvironment of LDH promote the increase of ^1^O2 quantum yield, which synergistically enhance the ability to tumor ablation of the nanohybrids both in vitro and in vivo. Shen et al.^[^ ^237^ ^]^ developed 2D CoMo‐LDH to highly active photosensitizer for near‐infrared PDT through defect engineering. The modified CoMo‐LDH nanosheets show greater reactive oxygen species production activity than the original CoMo‐LDH nanosheets under NIR‐III 1567 nm laser irradiation, successfully triggering cancer cell death in vitro and eliminating tumors in vivo. In addition, by hydrogen‐treating Ni‐Ti LDH films on NiTi surfaces, Yao et al.^[^ ^190^ ^]^ successfully fabricated Ni nanoparticle‐doped NiTiO_3_ films. According to the CCK‐8 studies, cells on LDH‐H_2_ samples only exhibited 20% viability under infrared radiation. In vivo studies showed the tumor stopped growing in the LDH‐H_2_ group after receiving infrared radiation, demonstrating the effectiveness of the photothermal impact of LDH‐H_2_ implants (Figure 13c).

Moreover, positively charged interfaces of LDHs preferentially interacted with negatively charged antitumor drugs and cell membranes for effective drug loading and delivery. Ray et al.^[^ ^238^ ^]^ fabricated methotrexate (MTX)‐encapsulated PLGA‐coated LDH nanoparticles (PLGA‐LDH‐MTX), which possess enhanced anti‐osteosarcoma efficiency. Higher MTX accumulation in target tumor tissues and suppressed tumor growth in tumor‐bearing mice were observed in PLGA‐LDH‐MTX group compared with bare MTX and PLGA‐MTX group. In addition, Oh et al.^[^ ^239^ ^]^ successfully used the conventional coprecipitation method to embed MTX into LDHs. In vitro anti‐cancer study revealed that MTX‐LDH hybrid, when used at a lower concentration, could exert the same impact as pure MTX. The potent anti‐cancer effect of MTX‐LDH composite lies in the effective and sustained delivery ability of LDHs for MTX, which may address the issue of adverse effects caused by excessive MTX dosage in real‐world applications. In another study, Chakraborty et al.^[^ ^240^ ^]^ demonstrate a fast one‐pot synthetic strategy involving simultaneous coprecipitation and in situ intercalation of MTX drug into the interlayer space of nanosized CaAl‐LDH to fabricate CaAl‐LDH‐MTX nanohybrid, which owns more potent cell inhibitory effects toward MG‐63 cells (92% cell inhibition) in comparison with that of free drug (80% cell inhibition). Overall, the much higher efficacy of LDH‐MTX compound compared with pristine drug is distinctly noticeable for a period of 72 h. In addition to delivering anti‐cancer drugs, targeting siRNA is also a strategy for treating tumors. Li et al.^[^ ^241^ ^]^ utilized mannose to couple siRNA into SiO2‐coated LDH nanocomposites, which effectively transmitted siRNA into tumor cells via receptor‐mediated internalization and significantly inhibited tumor cell growth.

## 7 LDHs for Analgesia

Pain is defined as unpleasant feelings that associated with tissue damage or potential tissue damage accompanied by negative emotional experiences.^[^ ^242^, ^243^ ^]^ As bone diseases are often associated with inflammation, trauma, and additional surgical tissue damage, pain is especially common among orthopedic disorders.^[^ ^244^, ^245^ ^]^ Nonsteroidal anti‐inflammatory drugs (NSAIDs) and glucocorticoids are the first‐line drugs for the treatment of bone disease‐associated pain in clinic.^[^ ^246^ ^]^ However, systemic use of NSAIDs or glucocorticoids can lead to adverse side effects including gastrointestinal reactions, cardiovascular risk, and endocrine disturbances.^[^ ^247^, ^248^, ^249^ ^]^ LDHs with effective loading and delivery abilities for both hydrophilic and hydrophobic drugs are expected to achieve firm binding as well as local, targeted, and sustained release of NSAIDs or glucocorticoids, so as to reduce systemic side effects and medication frequency.

### 7.1 LDHs Loaded with NSAIDs

**Figure 14:**
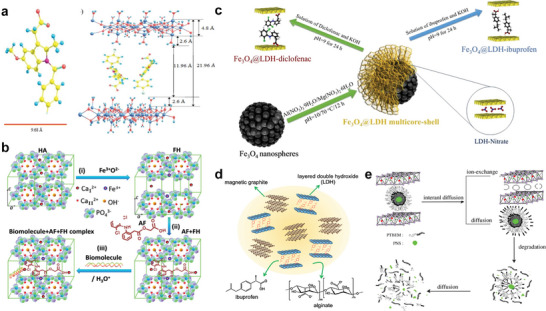
a) Molecular structure of indomethacin and spatial distribution structure of indomethacin after intercalation. Reproduced with permission.^[^ ^252^ ^]^ Copyright 2018, Taylor & Francis Group. b) Formation of LDHs structure of Fe‐induced hydroxyapatite, intercalation of AF drugs in LDH structures, and possible mechanisms of complex release of drugs. Reproduced with permission.^[^ ^253^ ^]^ Copyright 2016, Royal Society of Chemistry. c) Schematic diagram of preparation of Fe3O4@LDH multicore‐shell nanostructure. Reproduced with permission.^[^ ^254^ ^]^ Copyright 2020, Springer Nature. d) Diagrammatic presentation of LDH‐IBU magnetic bio‐nanocomposite structure. Reproduced with permission.^[^ ^257^ ^]^ Copyright 2014, Elsevier. e) Diagrammatic illustration for the drug release processes of PNS from PNS–LDH nanocomposite in a buffer solution. Reproduced with permission.^[^ ^263^ ^]^ Copyright 2009, Elsevier. AF, aceclofenac; IBU; ibuprofen; PNS, prednisone.

Apart from reducing adverse effects of systematic medication, the double‐layer structure of LDHs can protect the loaded NSAIDs from degradation or first‐pass elimination before being transported to orientated sites thus enhancing their pharmacological effects.^[^ ^250^ ^]^ NSAIDs encapsulated between the layers of LDHs can also effectively reduce the damage of drugs to gastric mucosa because of the anti‐acid property of LDHs.^[^ ^251^ ^]^ During the last decade, LDHs incorporated with different NSAIDs through various technologies such as ion exchange and coprecipitation have been widely researched. For instance, Soltani et al.^[^ ^252^ ^]^ synthesized a new organic/inorganic nanohybrid compound by simple anion exchange method for the intercalation of indomethacin into the interlayer of the Zn‐based LDHs (**Figure** **14**a). A series of characterization experiments confirmed that the nanocomposite possesses higher thermal stability and tends to reduce cell viability in a dose‐ and time‐dependent manner, making it an efficient drug deliverer for releasing hydrophobic drugs. Additionally, Manna et al.^[^ ^253^ ^]^ fabricated LDHs structure of Fe‐induced HA with different concentrations of Fe by in situ coprecipitation (Figure 14b). In this context, the newly developed LDHs were exceedingly biocompatible and capable to store and controlled release drugs in aqueous medium of phosphate buffer. Yousefi et al.^[^ ^254^ ^]^ fabricated a Fe3O4@LDH multicore@shell nanostructure as a novel pharmaceutical nanocarrier by a facile one‐step solvothermal route and coprecipitation experiment. In this case, ibuprofen (IBU) and diclofenac were successfully intercalated into the interlay space of LDHs by bridging bidentate interaction and both performed appropriate lipophilicity, water solubility, and steric effect under physiological conditions (Figure 14c). Also, Berber et al.^[^ ^255^ ^]^ intercalated naproxen (NP) and flurbiprofen (FB) into MgAl‐LDH via reconstruction and coprecipitation techniques to prepared NP‐LDH and FB‐LDH nanocomposites and found that the hydrophilic surface area of drug‐LDH composites increased the water penetration into the drug components. In the research of Bernardo et al.,^[^ ^250^ ^]^ MgAl‐LDH was successfully combined up to 80% w/w of NP by the structural reconstruction route. Pharmacokinetic studies showed a mild NP release, indicating the potentiality of MgAl‐LDH for local and sustained convey of naproxen at adequate concentrations. In addition, biological evaluation showed that the morphology and adhesion of cells at the administration site have not changed, demonstrating the biocompatibility and low cytotoxicity of MgAl‐LDH/NAP samples. These results describe a feasible measure for preparing effective systems for the local release of NSAIDs for biomedical applications. Moreover, Dagnon et al.^[^ ^256^ ^]^ conducted drug release studies with high performance liquid chromatography to ascertain the release profile of IBU from the nanocomposite films containing poly(l‐lactic acid) (PLLA) and IBU‐loaded ZnAl‐LDH. The staged elution release of the NSAIDs were observed, throwing light on the potential of LDH in promoting drug release abilities of biopolymers. Ribeiro et al.^[^ ^257^ ^]^ also prepared IBU as the LDH‐IBU intercalation compound to test the drug delivery efficiency. In this research, novel biohybrid magnetic matrices based on alginate and magnetic graphite nanoparticles was executed (Figure 14d). It was observed that owing to the additional physical barrier provided by inorganic layered host solids, the control of the IBU release rate has been particularly ameliorated. In addition, Willems et al.^[^ ^258^ ^]^ assessed a novel thermoreversible poly‐*N*‐isopropylacrylamide MgFe‐LDH (pNIPAAM‐MgFe‐LDH) hydrogel for intradiscal controlled delivery of the anti‐inflammatory drug celecoxib (CXB). In vitro results revealed that the release of CXB depends on the solubility of the hydrogel rather than the loading dose of CXB and CXB could suppress PGE2 production over a long period of time. Besides, the safety and effectiveness of CXB in the application of intervertebral discs has also been proved in vivo.

### 7.2 LDHs Loaded with Glucocorticoid

Glucocorticoids as classic anti‐inflammatory immunosuppressant and osteoinductive drugs possess outstanding analgesic effects.^[^ ^259^ ^]^ Since systemic glucocorticoid administration is associated with significant undesirable systemic side effects, local administration of glucocorticoid is typically preferred.^[^ ^260^, ^261^ ^]^ Unfavorably, the active form of glucocorticoids bears poor dispersion in physiological solutions thus is difficult for efficient dose delivery, for which LDHs with excellent hydrophobic drug loading and delivery abilities are expected to break the dilemma.^[^ ^262^ ^]^ Li et al.^[^ ^263^ ^]^ utilized poly(tert‐butyl acrylate‐*co*‐ethyl acrylate‐*co*‐methacrylic acid), an amphiphilic block copolymer, as building blocks for the preparation of negatively charged micelle containing hydrophobic drug prednisone, which was further intercalated into galleries of MgAl‐LDH for the study of pharmaceutical carrier and drug release behavior (Figure 14e). The results proved that in comparison with anomalous transport at pH 6.4 and the combination of diffusion through the matrix and degradation of the micelle at pH 4.8, this drug‐containing micelle intercalated LDH‐based composite exhibits complete and site‐specific drug delivery properties at pH 7.2 with a sustained release time up to 78 h. Kamyar et al.^[^ ^264^ ^]^ manufactured a novel nanohybrid material via intercalating dexamethasone phosphate into ZnAl‐LDH nanoparticles using the ion‐exchange methods. The successful intercalation of dexamethasone anions into ZnAl‐LDH was verified by XRD, FTIR, and STA. Besides, FTIR confirmed the successful entanglement of dexamethasone anions‐loaded ZnAl‐LDH with anodized titanium, where slower release rate was observed in ZnAl‐LDH/titanium compared to pristine titanium, indicating the excellent drug sustained release capability of LDHs.

## 8 Perspective and Conclusion

Although LDHs have been widely explored in treating bone diseases, considering their versatility, more potential applications of LDHs for improving the prognosis of bone diseases should be further studied. First of all, the application of LDHs in bone tissue engineering is underexploited. The current studies applying LDHs in bone tissue engineering mostly focus on the osteogenic properties of LDHs while ignoring the angiogenic and chondrogenic performances of LDHs. Similarly, angiogenic drugs and chondrogenic drug‐loaded LDHs for better tissue regeneration have not been reported in contrast to numerous studies focusing on osteogenic drug‐loaded LDHs. Considering the pioneering vascularization and chondrogenesis play an essential role in regulating subsequent bone regeneration,^[^ ^185^, ^265^, ^266^, ^267^ ^]^ the angiogenic and chondrogenic properties of LDHs should be further explored. In addition, nerve innervation has recently been reported to play critical roles in bone metabolism and regeneration processes.^[^ ^268^, ^269^ ^]^ Given many metal ions possessing considerable effects in promoting nerve formation and distribution, LDHs complexed with neurogenic metal ions should be designed and fabricated to promote neurogenesis for better bone regeneration.^[^ ^163^, ^270^, ^271^ ^]^

Second, with excellent biocompatibility and superior lubricating properties, LDHs reasonably hold great potential in treating osteoarthritis that characterized by increased friction coefficient between articular surface and chondrodegeneration, and are expected to replace the sodium hyaluronate products that widely used in clinic.^[^ ^106^, ^272^ ^]^ First, LDHs can form films attaching on articular cartilage surface thus reducing the friction coefficient in joint.^[^ ^72^ ^]^ Second, wearing cartilage with uneven surface can further accelerate cartilage abrasion, where the 2D LDHs nanosheets can automatically fill in the uneven sites in cartilage and smooth the cartilage surface to break the wretched cycle.^[^ ^273^, ^274^ ^]^ Third, the 2D LDHs tend to form a double‐layer “bearing‐like” construct in joint, which can convert sliding friction between cartilage surfaces into rolling friction thus greatly decrease cartilage wear.^[^ ^275^, ^276^ ^]^ In addition, the intrinsic chondrogenic properties and excellent drug delivery properties (analgesic, etc.) further highlight the prospects of LDHs in the comprehensive treatment of osteoarthritis. However, no research on LDHs as lubricating medium or drug delivery system for osteoarthritis treatment was reported, so further corresponding researches are urgently needed.

Third, LDHs and their nanocomposites as antitumor nanomaterials have been extensively exploited and achieved encouraging results based on the strategies of chemotherapy, immunotherapy, photothermal therapy (PTT), PDT, chemodynamic therapy (CDT), and sonodynamic therapy (SDT).^[^ ^49^, ^237^, ^277^, ^278^, ^279^ ^]^ However, for treating bone tumors, only LDHs‐based chemotherapy and PTT have been explored in the last decade.^[^ ^147^, ^190^ ^]^ For bone tumors hidden in deep tissue that are difficult for light to reach, so LDHs‐based PTT and PDT must overcome limited depth penetration of light sources and enhance the reactivity of nanomaterials to weak light for bone tumor treatment. By contrast, SDT and CDT that does not need light activation are comparatively promising for treating bone tumors and worthy of further study. For example, Hu et al.^[^ ^280^ ^]^ realized the phase transition of CoW‐LDH and NiW‐LDH nanosheets from polycrystalline to amorphous by simple acid etching treatment, as a high‐efficiency sonosensitizer for SDT. Due to the defect generation induced by phase transformation and electronic structure changes, amorphous CoW‐LDH nanosheets appear to have higher ROS‐generating ability under ultrasound irradiation and achieve effective tumor‐killing effects.

Fourth, the bioimaging and theranostics characteristics of LDHs have not been fully utilized in the treatment of bone diseases. Dynamic, real‐time, and non‐invasive monitor of the process of bone repair plays an important role in timely identifying poor bone alignment or healing thus improving the prognosis of bone fractures and defects.^[^ ^281^, ^282^, ^283^ ^]^ However, the most used imaging technology for skeletal system were X‐ray and computed tomography (CT), which can commonly imaging metal materials but not natural or synthetic polymers. Considering biodegradable polymers that do not require secondary removal surgery represent future advanced orthopedic implants, LDHs nanosheets containing metal ion and intercalated with various imaging contrast agents can enable the biodegradable polymers with outstanding radiopacity.^[^ ^284^, ^285^, ^286^ ^]^ Combined with the osteogenic and drug delivery properties of LDHs, multifunctional LDHs‐based orthopedic implants for theranostics are expected to be designed and fabricated. A pioneering study was conducted by Kim et al.,^[^ ^178^ ^]^ where the biodegradable poly(lactic‐*co*‐glycolic acid) (PLGA) bone plate was coated with RS‐loaded ZnAl‐LDHs and achieved favorable radiopacity under X‐ray and enhanced osteogenesis properties. Nevertheless, further studies exploring the bioimaging and theranostics characteristics of LDHs‐based nanocomposites for bone disease treatment are needed.

Fifth, with the development of transcriptome sequencing and single‐cell sequencing, increasing bone diseases have been identified to be associated with gene variation or abnormal transcription and translation, for which gene therapy seeking to modify or regulate gene expression or to manipulate gene ecology of cells for therapeutic application has attracted widespread attention.^[^ ^287^, ^288^, ^289^, ^290^, ^291^ ^]^ LDHs with great biocompatibility, adjustable interlayer structure, and protective confinement effect have been proved to be effective in gene loading and delivery. Tyner et al.^[^ ^292^ ^]^ introduced a full gene and promoter encoding green fluorescent protein (GFP) into the layers of LDH through simple, one‐step, ion exchange, which could minimize the amount of foreign DNA needed thanks to the protection of inorganic layers. As a result, the nanobiohybrids took effect in a variety of tissues as all cells internalizing and tolerating the nanohybrids. More importantly, all cells were able to express the gene and the transfection efficiency of some cell lines was up to 90%, showing a suitable approach for non‐viral gene vectors. In another work, LDH‐nucleic acid complexes were successfully formulated via encapsulating nucleic acids (pDNA, siRNA, and miRNA mimics and hairpin inhibitors) into MgAl‐LDH at varying mass ratios of LDH: nucleic acid. And in vitro transfection efficiency screening was carried out to assess cytocompatibility, cellular uptake, and functionality of LDH‐nucleic acid delivery to MSCs. According to the evaluation results, although the transfection of pDNA is not satisfactory, small, linear nucleic acids (siRNA, miRNA mimics, and hairpin inhibitors) were successfully delivered to MSCs with complexes exhibiting a favorable cytocompatibility profile and ideal function. Additionally, Costard et al.^[^ ^293^ ^]^ incorporated LDH‐miRNA nanoparticles into collagen‐nanohydroxyapatite scaffolds and confirmed the overexpression of miRNA in MSCs, suggesting the promising potential of LDH nanoparticles as delivery platform for gene therapy applications in regenerative medicine. Given the effective gene delivery abilities, LDHs‐based gene therapies are worthy to be explored to pursue efficient and precise treatment of gene disorder‐related bone diseases.

Finally, the potential of multifunctional LDHs in treating bone diseases remains to be explored. Orthopedic surgeries commonly face complex perioperative and intraoperative issues including bleeding control, analgesia, anticoagulation, and anti‐infection.^[^ ^294^, ^295^, ^296^, ^297^, ^298^ ^]^ The integrated management of these issues puts forward higher requirements for future advanced materials for orthopedic implants. The functional integration of thrombus and infection prevention, bleeding control, and postoperative pain management is expected to be designed based on versatile LDHs‐based nanocomposites for one‐stop treatment and integrated management of orthopedic surgery.

In conclusion, LDHs with adjustable metal ions composition and alterable interlayer structure possessing charming physicochemical characteristics, versatile bioactive properties, and excellent drug loading and delivery capabilities hold broad application prospects in the treatment of bone diseases. Despite great advances have been achieved, the potential of LDHs‐based nanomaterials for treating bone diseases is far from being fully explored and further studies are needed. Meanwhile, further studies are expected to pay more attention on the comprehensive biosafety evaluation of LDHs‐based nanomaterials of both implants and injectable complex forms. With the goal of facilitating clinical translation, the biodistribution, metabolic pathways, and degradation mechanisms of LDHs‐based nanomaterials, especially when the transition elements and rare earth metal ions are involved, should be fully studied and understood.

## Conflict of Interest

The authors declare no conflict of interest.
